# Multigram-Scale Asymmetric Alkene Reduction Catalyzed by a Thermostable Flavin Ene-Reductase

**DOI:** 10.1021/acs.oprd.5c00457

**Published:** 2026-05-13

**Authors:** Allison E. Wolder, Georg T. Höfler, Ombeline Mayol, Diederik J. Opperman, Frank Hollmann, Caroline E. Paul

**Affiliations:** † Biocatalysis Section, Department of Biotechnology, 2860 Delft University of Technology, Van der Maasweg 9, Delft 2629 HZ, Netherlands; ‡ Génomique Métabolique, Genoscope, Institut François Jacob, CEA, CNRS, Univ Evry, Université Paris-Saclay, 2 rue Gaston Crémieux, Evry 91057, France; § Department of Microbiology and Biochemistry, 37702 University of the Free State, Bloemfontein 9300, South Africa

**Keywords:** biocatalysis, old yellow enzymes, scale-up, monoterpenes, *E*-factor, ene-reductase

## Abstract

Chiral-substituted carbonyl compounds can be sustainably obtained via asymmetric alkene reduction catalyzed by ene-reductases from the Old Yellow Enzyme (OYE) family. Yet OYEs are seldom implemented in scale-up reactions due to low turnover numbers (TONs). Herein, we demonstrate multigram 150 g/L scale reactions with the thermostable OYE from *Thermus scotoductus* (0.2 wt %) for the asymmetric reduction of monoterpenes. Best results were achieved with (*S*)-carvone (7.5 g), affording a record TON of 123,000, with 90% isolated yield and >99% enantiomeric excess of (2*R*,5*S*)-dihydrocarvone, and an environmental *E*-factor of 11.6.

## Graphical abstract

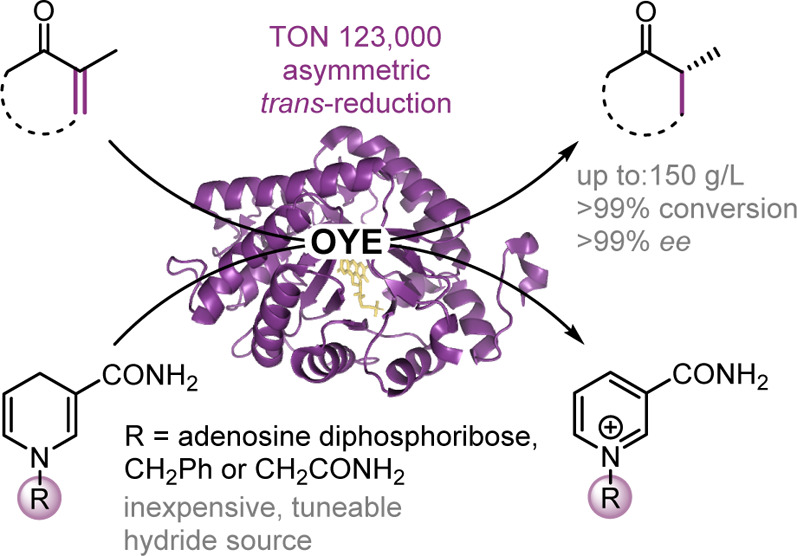

## Introduction

Asymmetric reduction of alkenes provides access to valuable chiral-substituted alkanes, essential building blocks for commercial pharmaceutical drugs,^1^ agrochemicals, and fine chemicals such as fragrances.^2,3^ Traditionally, asymmetric reduction is carried out via hydrogenation with hydrogen gas and metal catalysts (Pd, Pt, Ni, Rh, Ru, Ir), which can be costly, toxic, and lacking in selectivity to reach the >99% enantiomeric excess (*ee*) usually desired.^1,4^ In particular, homogeneous catalysts Rh and Ir with chiral ligands, the industrial go-to for asymmetric hydrogenation, require high loadings (1–4 mol %) and low to high hydrogen gas pressure (1–50 bar), display stability issues, and are prematurely disposed due to high costs for precious metal recovery, leading to questions of sustainability (Figure 1a).^5^

**Figure 1:**
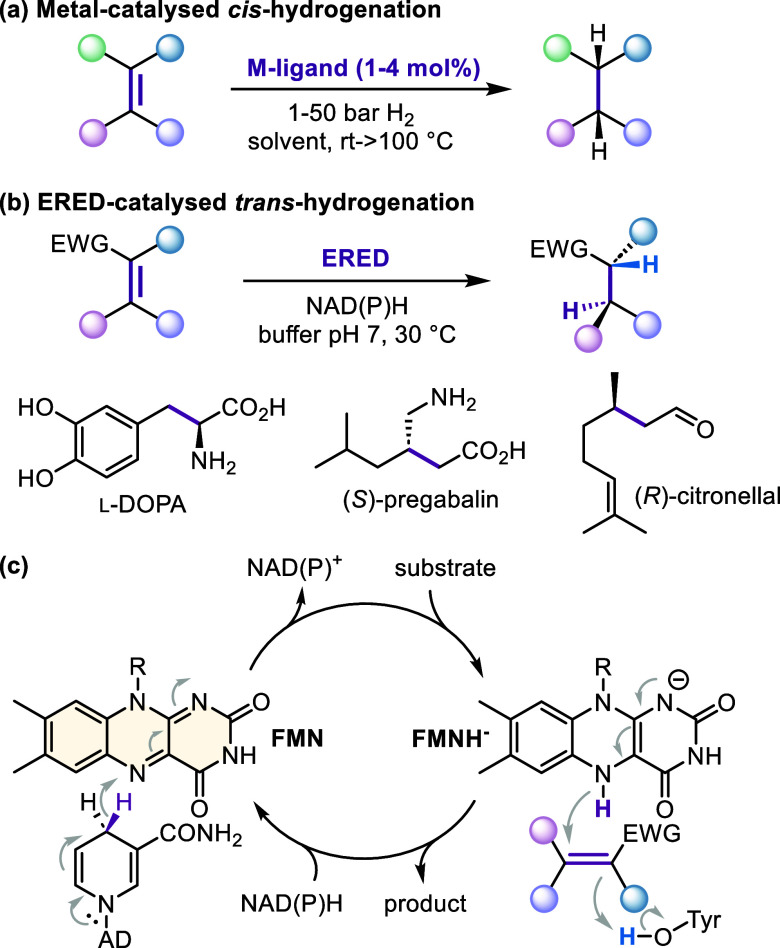
(a) Metal-catalyzed hydrogenation. (b) ERED-catalyzed asymmetric reduction of activated alkenes, examples of chiral products that can be obtained. (c) Simplified ERED bi-bi ping-pong mechanism. EWG = electron-withdrawing group, e.g., carbonyl, nitro, and nitrile.

Developing biocatalysts for industrial-scale asymmetric reduction of alkenes is an attractive alternative^6,7^ due to their exquisite high selectivity and activity, requiring only low loadings, due to mild reaction conditions, and providing shorter synthetic routes.^8–11^ Biocatalysts such as ene-reductases (EREDs) catalyze the asymmetric reduction of activated alkenes to generate products with up to two chiral centers (Figure 1b).^12^ Small-scale laboratory studies already showed that EREDs from the Old Yellow Enzyme family (OYE, EC 1.6.99.1) are excellent candidates for industrial processes.^2,3,13–15^ OYEs contain a noncovalently bound prosthetic flavin mononucleotide FMN, which acts as an electron mediator, and require a β-nicotinamide adenine dinucleotide NAD­(P)H cofactor to provide a hydride. The reaction follows a bi-bi ping-pong mechanism wherein NAD­(P)H reduces FMN and NAD­(P)^+^ dissociates to allow the alkene substrate to enter and be reduced in a *trans* addition fashion at its β-carbon by FMNH^–^ and protonated at its α-carbon by a tyrosine residue in close proximity (Figure 1c), thus affording the chiral product.^12^

Most OYE-catalyzed reactions have been carried out so far with rather low substrate concentrations, mainly due to screenings of substrate scope.^12,16^ Only a handful of OYE-catalyzed gram-scale reductions of alkenes have been performed (Table 1),^17–25^ such as for geranial (*E*)-**1a**, citral (*E*/*Z*)-**1a**, (2*E*)-decenal **2a**, 1-acetylcyclohexene **3a**, dimethyl itaconate **4a**, methyl 3-oxocyclohexene-1-carboxylate **5a**, (*R*)-carvone (*R*)-**6a**, (*S*)-carvone (*S*)-**6a**, and ketoisophorone **7a**. However, these biocatalytic reactions fell within rather low turnover numbers (TONs) of 10^3^–10^4^ and even in some cases <1000 when using whole cells (entry substrate **5a**).

**Table 1:**
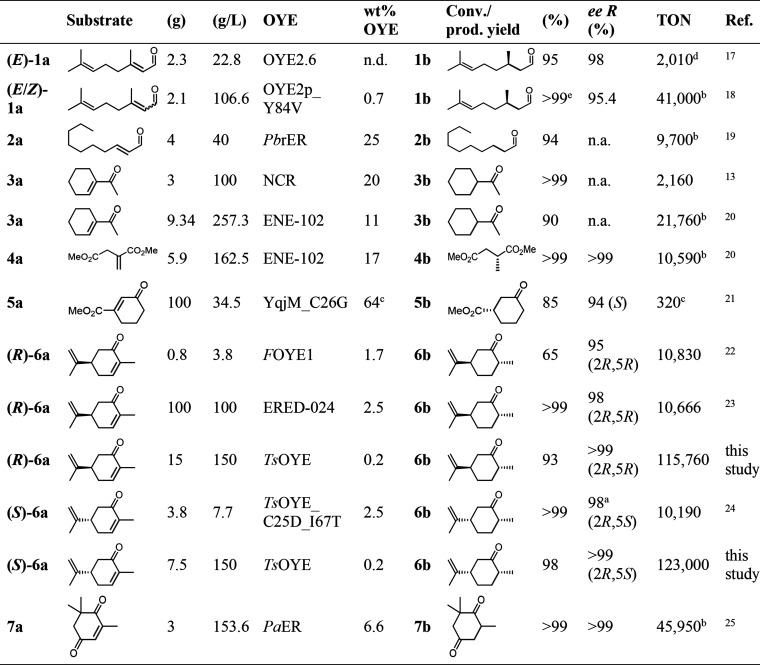
Comparison of Gram-Scale ERED-Catalyzed Asymmetric Alkene Reductions^a^ ^,^ ^b^ ^,^ ^c^ ^,^ ^d^ ^,^ ^e^

a Includes 2% enantiomer from the starting material.

b Calculation with an estimated 10% OYE in the lysate.

c Calculation assuming 10% YqjM_C26G present in whole cells added as 640 wt %.

d Calculation assuming crude enzyme activity was 1.75 U/mg, wherein 100 U was used with 15 mM substrate in a 100 mL volume reaction.

e 64% isolated yield; n.d. = not defined; n.a = not applicable.

Research into optimization and scale-up of enzymatic reactions is necessary,^23,26^ as often, biocatalytic processes are viewed as being too expensive to be economically viable and having a long optimization process.^27^ The current guiding principles for an industrially feasible biocatalytic scale-up are to achieve, within 24 h, >95% conversion with >99.5% enantiomeric excess (*ee*), at >100 g/L substrate while having a substrate-to-enzyme ratio of >50, an enzyme loading weight percent (wt %) of <2 ^16^ and minimizing the concentration of cofactor to <0.5 g/L.^28^ We set out to reach the described standards, as well as >10^4^ TONs, using the biocatalyst *Thermus scotoductus* Old Yellow Enzyme (*Ts*OYE). *Ts*OYE is a robust, thermostable ERED, catalyzing the reduction of several alkenes with high activity.^24,29,30^ Recent immobilization of *Ts*OYE on Celite to use in organic solvents such as methyl *tert*-butyl ether (MTBE) with <10% aqueous medium also shows its robustness in such a medium.^31^

Regarding the required cofactor, a glucose dehydrogenase (GDH)-catalyzed recycling system has demonstrated its efficiency and can be used for either NADH or NADPH *in situ* regeneration.^32,33^ The use of alternative synthetic nicotinamide coenzyme biomimetics (NCBs) could potentially reduce costs and simplify the process even further.^34–36^ A 1-benzyl-1,4-dihydronicotinamide (BNAH) cofactor analogue and 1-(2-carbamoylmethyl)-1,4-dihydronicotinamide (AmNAH) are seemingly easy to synthesize^37,38^ and can kinetically perform as well as or even better than natural cofactors to reduce flavin systems.^39–41^ Yet, ideally, the NCBs should be recycled to be cost-effective.^35,42^

In this study, we demonstrate multigram-scale asymmetric alkene reduction catalyzed by *Ts*OYE with different cofactor systems. A model substrate, the monoterpene carvone, was reduced to dihydrocarvone (Table 1), a valuable chiral precursor for several natural products and pharmaceutical drugs.^22,23^ We studied the influence of cosolvents, substrate concentrations up to 150 g/L, enzyme concentration, TON, cofactor stability, and recycling, with the overall environmental *E*-factor to evaluate sustainability. We also developed *in situ* NMR monitoring of the OYE-catalyzed reaction.

## Results and Discussion

We could produce and heat-purify *Ts*OYE as previously described, on a 1 L as well as 15 L scale, obtaining 2.4 g of pure active enzyme fully saturated with FMN (see Supporting Information Figure S1). The measured specific activities for the classic model substrate cyclohexenone as well as (*R*)-carvone at different temperatures and pH (Supporting Information Table S5), showed *Ts*OYE to be most active at temperatures up to 65 °C and at pH 8.0. Cosolvents such as dimethyl sulfoxide (DMSO), isoamyl acetate (IAA), and acetone were tested to aid substrate solvation in aqueous buffer with 10–200 mM (*R*)-carvone (Supporting Information Figures S9 and 10). Interestingly, the highest activity was obtained with 20% (v/v) DMSO, in contrast with IAA and acetone or no cosolvent. These results correlate with those of Kroutil and co-workers,^43^ where *Ts*OYE activity increased with up to 30% v/v DMSO for cyclohexenone reduction. We performed size exclusion chromatography on *Ts*OYE with and without 30% v/v DMSO and observed no change in the oligomeric state (Supporting Information Figure S2). This implies DMSO does not influence the latter and may in fact help with substrate and product solvation. Increasing to 50% v/v DMSO reduced both specific activity and conversion by half (Supporting Information Figure S10).

Running biocatalytic reactions with a 10–200 mM substrate and a higher buffer strength of 200 mM at 40 °C improved conversion (Figure 2). Reactions of 1 h with 50 mM (*R*)-carvone gave 86% conversion in 200 mM MOPS versus only 48% conversion in 50 mM MOPS (Supporting Information Figure S11B), both affording >99% diastereomeric excess (*de*) of (2*R*,5*R*)-dihydrocarvone. This effect can be ascribed to the basification of the reaction due to the consumption of protons when using BNAH as a cofactor. However, when comparing specific activities, higher salt concentrations can have a negative influence on *Ts*OYE activity; therefore, buffer strength should be carefully evaluated.^30^ The temperature increase from 30 to 40 °C had more impact at higher concentrations, 100 and 200 mM (*R*)-carvone, enabling up to 30,000 TON (Figure 2).

**Figure 2:**
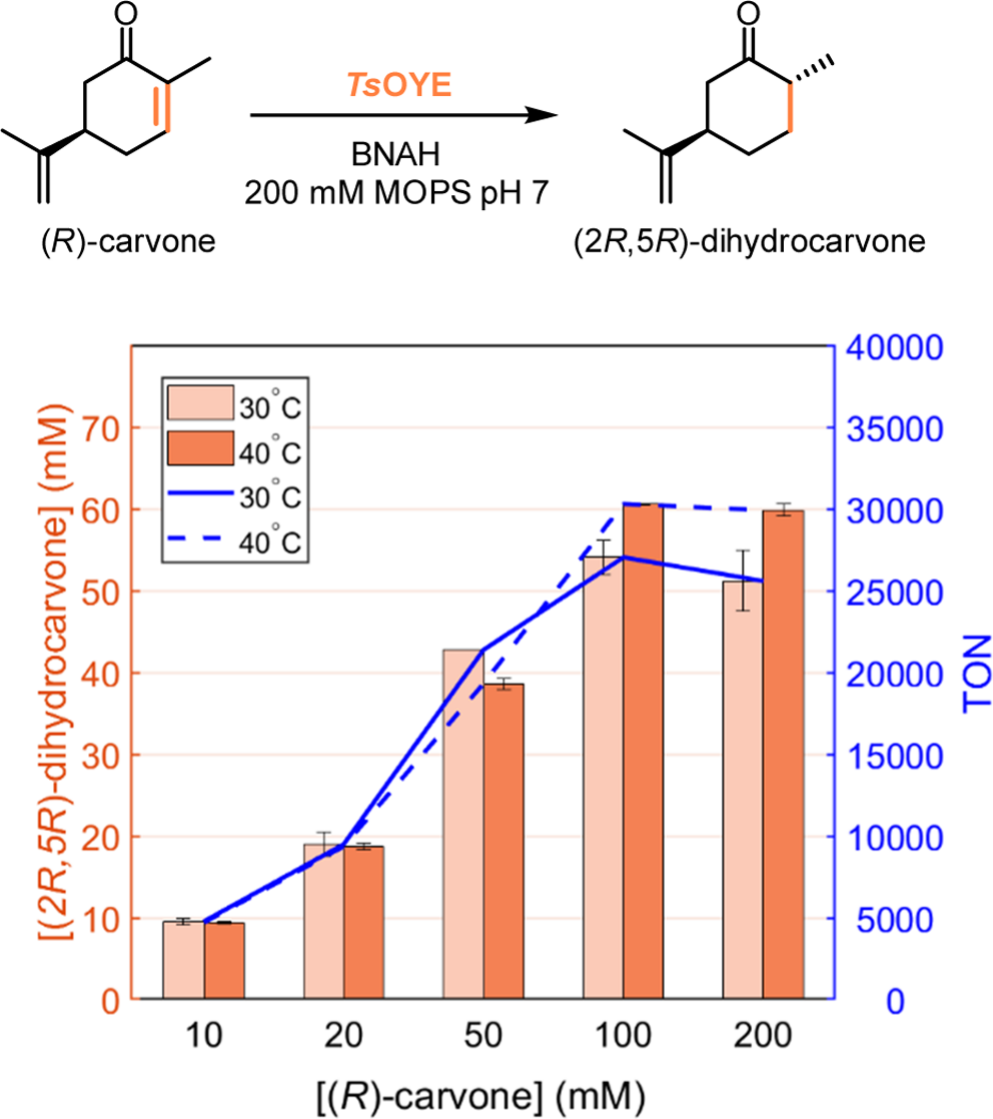
Influence of substrate concentration and temperature on *Ts*OYE-catalyzed (*R*)-carvone reduction with BNAH at 30 and 40 °C. Conditions: 1 mL volume, 200 mM MOPS-NaOH pH 7, 1, 2, 4, 8, and 17% v/v DMSO for 10, 20, 50, 100, and 200 mM (*R*)-carvone, respectively, 10% excess [BNAH] with respect to [(*R*)-carvone], 0.03–0.7 wt % (2 μM) *Ts*OYE, 900 rpm, 1 h. Average of duplicates.

We compared an NADPH recycling system using GDH and glucose (Supporting Information Figure S14) with synthetic cofactors BNAH and AmNAH (Supporting Information Figure S12). Starting from 200 mM (*R*)-carvone, BNAH gave the highest conversion (66%), followed by AmNAH (50%), and then last by GDH recycling of NADPH (29%). The pH of the reactions prior to extraction was also estimated using pH paper. In general, reactions with BNAH ended at pH ∼9 and GDH recycling at pH ∼6. The concomitant conversion of glucose to gluconolactone, which hydrolyzes to gluconic acid, acidifies the reaction medium when using the NADPH recycling system.

Enzyme loading was explored and increased from 0.03 to 0.3 wt % (2–16 μM) *Ts*OYE. Initial results showed that for ≥4 μM enzyme, 200 mM (*R*)-carvone, reactions with BNAH after 1 h leveled off with a maximum of 120 mM of product, giving a turnover frequency TOF of <30,000 h^–1^ (Supporting Information Figure S12A). However, reactions with AmNAH needed 4 h to reach the same product maximum of 120 mM, TOF <7,500 (Supporting Information Figure S12B). The reactions were repeated over a 24 h time frame (Figure 3). Stoichiometric addition of BNAH and AmNAH greatly increased the viscosity of the reaction compared to the GDH/glucose recycling system. BNAH outperformed both AmNAH and the GDH recycling system, with the lowest enzyme concentration (2 μM *Ts*OYE), performing with a TON of 88,895 and 89% conversion (ca. 180 mM product). The poorer performance of the GDH recycling system was ascribed to the acidification of the reaction, even with 200 mM MOPS buffer. However, product formation reached a plateau at 200 mM in 24 h using BNAH, regardless of starting with 200, 400, or 800 mM (*R*)-carvone (Supporting Information Figure S13A). Feeding the reaction with BNAH in increments improved conversion, achieving up to 400 mM product in 72 h (Supporting Information Figure S13B,C). The absence of DMSO as a cosolvent resulted in lower conversions overall (Supporting Information Table S6).

**Figure 3:**
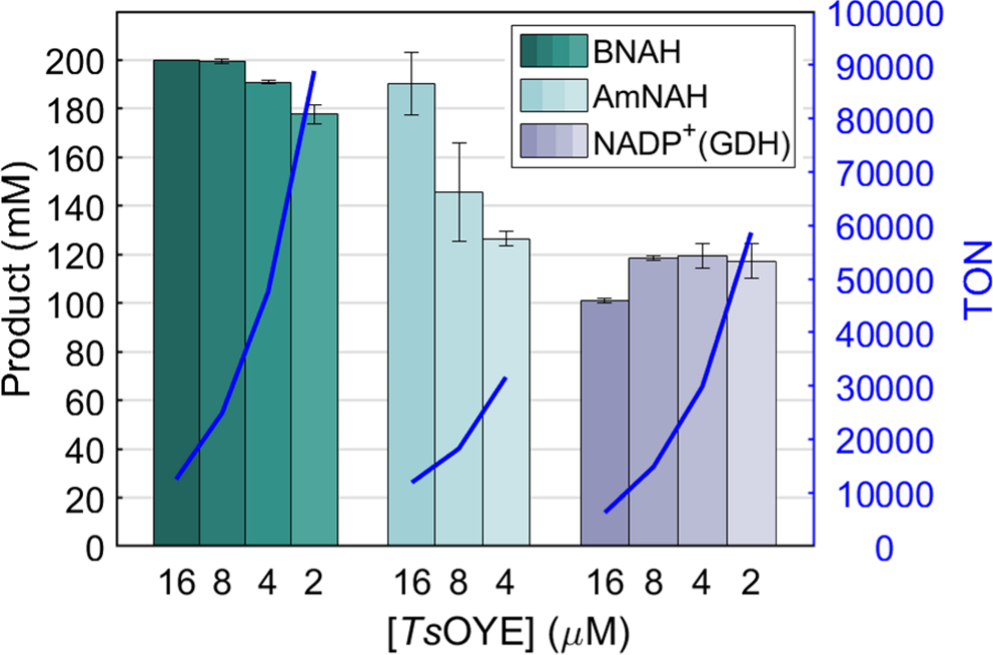
Influence of the cofactor and enzyme concentration on *Ts*OYE-catalyzed (*R*)-carvone reduction. Conditions: 1 mL volume, 200 mM MOPS-NaOH pH 7, 200 mM (*R*)-carvone, 17% v/v DMSO, 30 °C, 900 rpm, 24 h. Cofactors BNAH and AmNAH with 10% excess (220 mM), GDH cofactor recycling with 200 mM glucose, 3 U/mL *Bs*GDH, 0.1 mM NADP^+^, 0.03–0.3 wt % (2–16 μM) *Ts*OYE. Average of duplicates.

Cofactor stability between NADPH, NADH, and synthetic cofactors BNAH and AmNAH was measured in different buffers and pH (Figure 4). Phosphate buffer clearly accelerated decomposition of all reduced cofactors, especially at acidic pH and high buffer concentrations, as previously reported for NAD­(P)­H.^44^ MOPS buffer at pH 7 gave a slower decomposition rate, and Tris buffer clearly showed the slowest rate. Overall, NADH was the most stable, and AmNAH was more stable than BNAH and NADPH. Therefore, when using reduced cofactors, acidic and phosphate buffers should be avoided, and Tris buffer should be preferred.

**Figure 4:**
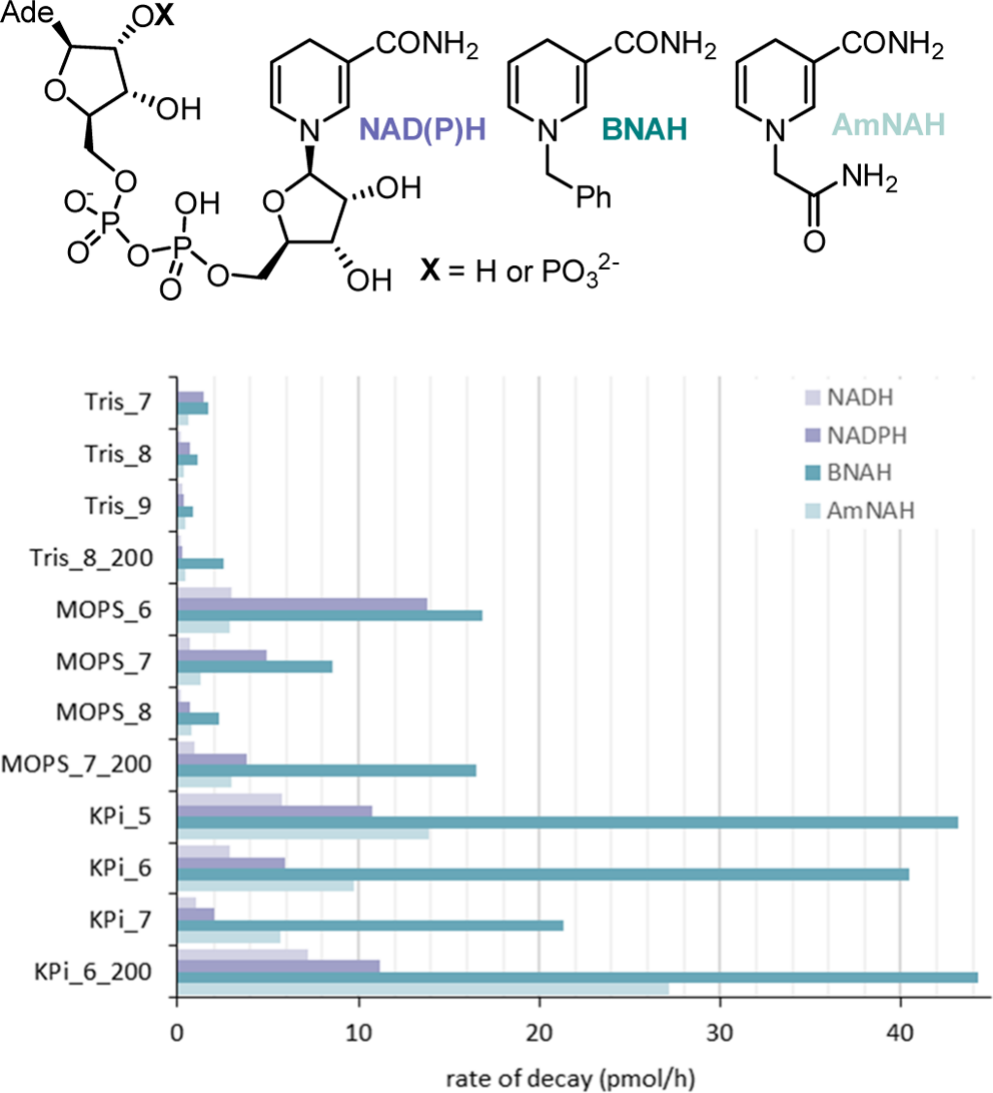
Cofactor stability study for NADPH, NADH, BNAH, and AmNAH in 50 or 200 mM Tris, MOPS, and KPi at pH 5–9, measured by UV–vis spectroscopy over 5 h at 30 °C (see Supporting Information).

To mitigate the change of pH, the multigram scale-up reactions with 1 M substrate were carried out with a pH dosimeter using either acid (1 M HCl) with BNAH, or base (2 M NaOH) with GDH/glucose recycling, in 200 mM Tris–HCl buffer to maintain pH at 8 (Supporting Information Figure S16). A scale-up with 1 M (*R*)-carvone was carried out in a 100 mL volume using BNAH (Supporting Information Figure S16A). The reaction was run at 40 °C with BNAH added in increments and reached 85% conversion after 72 h with a TON_*Ts*OYE_ of 106,120. The same reaction was carried out with a GDH/glucose recycling system, achieving even better results with 98% conversion and a TON of 115,765. The lower conversion with BNAH compared to NADPH recycling may be due to the lack of stability of BNAH over time. Finally, 1 M (150 g/L) (*S*)-carvone was also reduced (Figure 5), giving 98% conversion, 123,000 TON, with 6.9 g (90%) isolated yield of (2*R*,5*S*)-dihydrocarvone with >99% *ee*.

**Figure 5:**
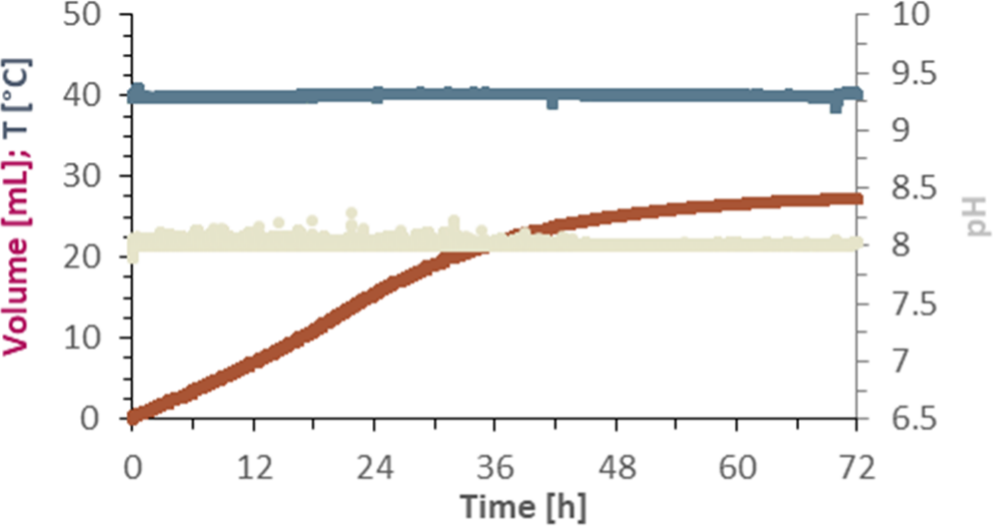
Scale-up of *Ts*OYE-catalyzed reduction of 1 M (*S*)-carvone in a 50 mL volume. Conditions: 200 mM Tris–HCl pH 8.0, 1.1 M glucose, 3 U/mL *Bs*GDH, 1 mM NADP^+^, 0.2 wt % (8 μM) *Ts*OYE, 40 °C, pH control with 2 M NaOH. 98% conversion, 90% isolated yield of 6.9 g (90%) of (2*R*,5*S*)-dihydrocarvone, 99% *de*. TON based on conversion: 123,000.

We calculated the environmental *E*-factor,^45^ a metric used to evaluate the environmental impact of manufacturing, by measuring the total mass of waste produced (including base addition) to obtain the final compound of this scale-up reaction. The *E*-factor was found to be 11.6 (kg of waste/kg of product), with water and base accounting for 59% and 29% of the mass waste, respectively. This value is relatively low for the fine chemical and pharmaceutical industry, also considering no toxic metals are involved, and the extracted isolated product is already of high (∼98%) purity (Supporting Information Figure S20). Diethyl ether was used as the preparative scale extraction solvent due to its low boiling point and good solubility; however, greener solvent alternatives such as 2-methyltetrahydrofuran could be employed.

^1^H NMR spectroscopy was also used to measure bioconversions *in situ* and to observe whether any intermediates were formed. NMR may have a higher limit of detection than that of GC or HPLC, yet it offers real-time measurements in a closed system, in which reactant and product concentrations remain constant. Applications of the measurements could be as vast as deducing reaction mechanisms, calculating reaction rates, following pH changes, or discovering fleeting intermediate species or unknown byproducts normally lost through extraction.^46^ In a first set of NMR experiments (see Supporting Information NMR *in situ* monitoring section for details), we determined the reaction conditions that would allow reproducible peak integration of the substrate and product, ensuring that each was positioned either upshield or downshield relative to the water peak, which through different pulse programs and settings was suppressed. After optimization and calibrations (Supporting Information Figures S21–27), we measured the *Ts*OYE-catalyzed reduction of 50 mM (*R*)-carvone and cyclohexenone using the presat PURGE program (Figure 6). The decrease and increase of the selected proton peaks could be followed.

**Figure 6:**
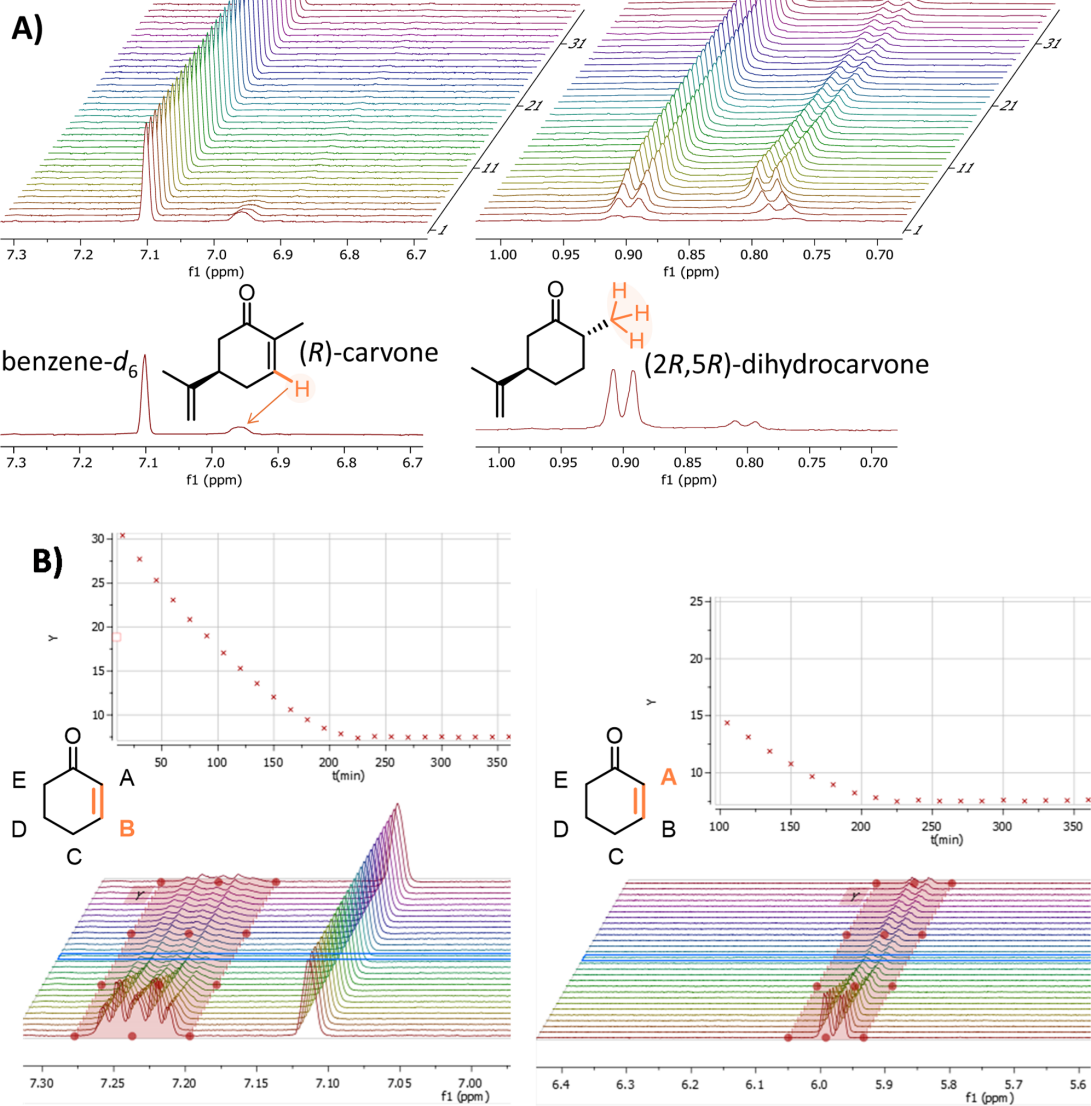
^1^H NMR monitoring of *Ts*OYE-catalyzed reactions. (A) 50 mM (*R*)-carvone reduction. Two peaks were monitored: (*R*)-carvone singlet peak (∼6.95 ppm) and (2*R*,5*R*)-dihydrocarvone doublet peak (∼0.90 ppm). The standard C_6_D_6_ peak appears at 7.10 ppm. Conditions: 200 mM KPi–HCl buffer pH 7.0, 3 U/mL *Bs*GDH, 55 mM glucose, 0.1 mM NADP^+^, 2 mg (50 mM) (*R*)-carvone, 4% v/v DMSO, 1 wt % (2 μM) *Ts*OYE (300 μL volume). Presat PURGE pulse sequence, 16 scans, measured every 5 min. See Figures S28 and S29 for details. (B) 50 mM cyclohexenone reduction, same conditions. See Figures S30 and S31 for details.

Figure 6A NMR overlaid spectra give an overview of two main peaks, as well as the standard deuterated benzene at 7.16 ppm. The decreasing singlet peak at 6.98 ppm represents the β-carbon proton of substrate (*R*)-carvone, while the increasing doublet peak at 0.92 ppm represents the methyl group protons on the α-carbon of the product (2*R*,5*R*)-dihydrocarvone. A product peak appearing at 0.8 ppm may relate to the enolization of dihydrocarvone. We also suspect delocalization of bound and unbound states of the product occurs, as some peaks showed a chemical shift over time (Figure 6A, see Supporting Information Figures S28 and S29). In Figure 6B, the array spectra show a clear decrease of the cyclohexenone double bond proton peaks at 7.25 and 6.0 ppm. Compared with the experiment with 50 mM (*R*)-carvone, the 50 mM cyclohexenone reduction reaction showed fewer signs of diffusion due to better solubility in aqueous buffer and displayed full conversion. Overall, this NMR method could be used to observe potential intermediate species and could potentially be performed under anaerobic conditions for other sensitive OYE-catalyzed reactions such as C–C bond formation.^47^ Diffusion limitations, however, may prevent it from scale-up monitoring of high substrate loading (Supporting Information Figure S24) unless a cosolvent is used.

## Conclusions

In summary, the OYE can be applied for scale-up asymmetric alkene reduction of 150 g/L monoterpenes. Best results were obtained with (*S*)-carvone (7.5 g) using 0.2 wt % *Ts*OYE and reaching 123,000 TON to access valuable chiral carbonyl compounds >99% *ee*, with an overall environmental *E*-factor of 11.6. *Ts*OYE, especially compared to other OYEs, has the potential to be scaled up even further. The ability for OYE to be used in cascades enables tunability for scale-up reactions toward further functionalized products such as chiral alcohols^23,48,49^ and amines.^30,50^ OYEs can also perform well in organic solvents^31,43,51^ and thus be used to reduce water-sensitive substrates. These reactions could potentially be monitored *in situ* by NMR spectroscopy and enhance their mechanistic understanding, which is currently under further investigation.^47^

## Experimental Section

### Activity Assay

*Ts*OYE activity was determined by UV–vis measurements of NADPH consumption at 340 nm (ε = 6.22 mM^–1^ cm^–1^) in various conditions (30, 40, and 65 °C, different buffers, and cosolvents). Glucose oxidase (GOx) and glucose were added to consume molecular oxygen. Measurements were executed in duplicate.

### Enzyme Production

*Ts*OYE was recombinantly produced in*E. coli* as previously reported.^29,39^ The cells were harvested by centrifugation (10,000 rpm, 4 °C, 20 min), resuspended and washed in MOPS-NaOH buffer (20 mM, pH 7.0), and centrifuged (10,000 rpm, 4 °C, 20 min). The cell pellets were then resuspended in MOPS-NaOH buffer (20 mM, pH 7.0) and lysed using a multicycle cell disruptor (Constant Systems). The soluble fraction was obtained after centrifugation (10,000 rpm, 4 °C, 20 min). The cell-free extract was heat-purified (70 °C, 90 min) and centrifuged (8,000 rpm, 4 °C, 30 min), and the supernatant was incubated with excess FMN overnight at 4 °C. The solution was concentrated with an Amicon 30 kDa and passed through a PD-10 desalting column into buffer MOPS-NaOH (50 mM, pH 7.0) and stored at −70 °C.

### Biocatalytic Reactions

#### Analytical Scale

Various concentrations (0.1–0.8 M or 15–120 mg) of (*R*)-carvone in 1 mL volume were screened, with the cosolvent DMSO, IAA, or acetone, with buffer (50 or 200 mM) MOPS-NaOH pH 7.0 or 200 mM KPi pH 7.0, with the GDH cofactor system (0.1 or 0.2 mM NADP^+^, 100–220 mM glucose, 10 mg/mL GDH from Evocatal, or 3–9 U/mL *Bs*GDH) or synthetic cofactor (10% excess of stoichiometric amounts BNAH or AmNAH), and *Ts*OYE, in an 2 mL Eppendorf tube, at 30 °C, 700 rpm, and various times. The reaction was quenched by extraction with 0.5 mL of ethyl acetate, vortexed, centrifuged (13,000 rpm, 2 min), and dried with MgSO_4_.

#### Preparative Scale

There were no significant hazards or process risks for scale-up. The reactions were performed in a 250 mL 3-neck round-bottom flask, with a 50 or 100 mL starting volume, with continuous top stirring. Added to the reaction was substrate (*R*)-carvone or (*S*)-carvone (7.5 g in 50 mL or 15 g in 100 mL, 1 M), with Tris–HCl (200 mM, pH 8.0) or MOPS-NaOH (200 mM, pH 7.0) buffer, with a GDH cofactor system (0.2 mM NADP^+^, 1–1.2 M glucose, 3–12 U/mL *Bs*GDH) or synthetic cofactor BNAH (14.2 g or 1.3 M), 0.2 wt % (8 μM) *Ts*OYE. The pH was maintained with 2 M NaOH (GDH system up to pH 7–8) or 1 M HCl (BNAH system up to pH 8). The reaction was performed in the dark (covered with aluminum foil) and maintained at 40 °C by submerging the flask in a heat-controlled stirred water bath. The product was extracted with three times ∼75 mL of diethyl ether.

### GC Analyses

Column Lipodex E (Macherey-Nagel) 50 m × 0.25 mm × 0.25 μm; split ratio 100; linear velocity 38 cm/s; helium; method: 80 °C for 2 min, 5 °C/min until 110 °C and hold for 5 min, 5 °C/min until 130 °C and hold for 5 min, 20 °C/min until 220 °C and hold for 1 min. Retention times: 14.9 min (2*S*,5*S*)-dihydrocarvone, 15.2 min (2*R*,5*R*)-dihydrocarvone, 16.4 min (2*S*,5*R*)-dihydrocarvone, 18.0 min (*R*)-carvone/(*S*)-carvone.

## Supplementary Material



## Notes

Enzyme production, cofactor synthesis, biocatalytic conversions, NMR method, and spectra (PDF)
