# Supplementary material for: Multigram-Scale Asymmetric Alkene Reduction Catalyzed by a Thermostable Flavin Ene-Reductase
Source: Org Process Res Dev. 2026 May 13;30(6):1495–502. doi: 10.1021/acs.oprd.5c00457 (PMC13288856; doi:10.1021/acs.oprd.5c00457)
Supplement: Supplementary file 1 [file op5c00457_si_001.pdf]

Supporting information

Multigram scale asymmetric alkene reduction catalyzed by a thermostable flavin ene-reductase

Allison E. Wolder,[a] Georg T. Höfler,[a] Ombeline Mayol,[b] Diederik J. Opperman,[c] Frank Hollmann,[a] and Caroline E. Paul*,[a]

[a] *Biocatalysis section, Department of Biotechnology, Delft University of Technology, Van der Maasweg 9, 2629HZ Delft, The Netherlands; c.e.paul@tudelft.nl*

[b] *Génomique Métabolique, Genoscope, Institut François Jacob, CEA, CNRS, Univ Evry, Université Paris-Saclay, 2 rue Gaston Crémieux 91057 Evry, France*

[c] *Department of Microbiology and Biochemistry, University of the Free State, Bloemfontein 9300, South Africa*

Contents

General information 2
Chemicals 2
Cofactors 2
Enzymes 2
Analytic equipment 2
Enzyme production and purification 3
*Ts*OYE 3
Activity assays 3
Oligomerization state 4
Turnover calculations 4
Cofactor stability study 5
Gas chromatography analyses 7
*Ts*OYE-catalyzed bioreduction reactions 10
Analytical scale 10
With GDH-catalyzed NADPH cofactor recycling 10
With synthetic cofactors 10
Preparative scale 10
Supplementary figures 11
10-200 mM (*R*)-carvone 12
200-800 mM (*R*)-carvone 14
With synthetic cofactors BNAH and AmNAH 14
With GDH cofactor recycling 15
800 mM - 1 M carvone 16
10 mL volume 16
50-100 mL volume 16
NMR *in situ* monitoring 20
(*R*)-Carvone reduction 21
Cyclohexenone reduction 29
Reaction set-up 29
References 31

General information

Chemicals

All commercial reagents and solvents were purchased with the highest purity available and used as received. Specifically, the following chemicals (Chemical Abstract Service CAS number in brackets) were obtained from Merck Sigma-Aldrich: (*R*)-(–)-carvone (98%, 98% *ee*, 6485-40-1), (*R*,*S*)-dihydrocarvone (mixture of isomers, 7764-50-3), (*S*)-(+)-carvone (96%, 2244-16-8), (–)-dihydrocarveol (≥95% with isomers, 20549-47-7), cyclohexenone (>98%, 930-68-7), cyclohexanone (≥99.5%, 10894-1), D-(+)-glucose monohydrate (14431-43-7), ethyl acetate (EtOAc, ≥99.5%, 141-78-6), dodecane (112-40-3), isoamyl acetate (IAA, ≥95%, 123-92-2), nicotinamide (98-92-0) and 2-chloroacetamide (79-07-2). Dimethyl sulfoxide (DMSO, 6768-5) and acetonitrile (MeCN, 75-05-8) were obtained from VWR International (Radnor, PA, USA).

Deuterium oxide (D_2O, 99.9%, 7789-20-0) and deuterated chloroform ($CDCl_3$, 99.8%, 865-49-6) were purchased through Eurisotop (Saint-Aubin, France). Deuterated benzene (C_6D_6, 99.5%, 1076-43-3) was obtained from Cambridge Isotope Laboratories Inc.

Enantiopure (6*R*)-levodione was provided by Dr. A.J.J. Straathof, produced from yeast fermentation (Department of Biotechnology, TU Delft).[1]

Cofactors

β-$NADP^+$ (sodium salt hydrate, 97.6% purity, 4.8% water content, 698999-85-8) was obtained from Prozomix (Haltwhistle, Northumberland, UK).

β-NADPH (tetrasodium salt, ≥93, <8% water content, 2646-71-1) was purchased from the Oriental Yeast Co. (OYC EU, Rotterdam, The Netherlands).

Artificial cofactors BNAH, CNNAH, BAPH, and AmNAH had been synthesized and purified as previously described on a 30 g scale.[2-4]

Enzymes

Glucose oxidase from *Aspergillus niger* (GOx, 9001-37-0) was purchased from Sigma Aldrich.

Lyophilized powder glucose dehydrogenase GDH (specific activity 35 U/mg) used for initial screenings, was obtained from a kit from Evocatal (now Evoxx technologies GmbH, Monheim am Rhein, Germany).

The thermostable GDH E170K_Q272L double mutant from *Bacillus subtilis* strain 168 (*Bs*GDH) was used for intensification and scale-up reactions, recombinantly produced in *E. coli* as previously described.[5] A 15-L fermentation produced 500 g wet cells, and an estimated 6.8 g of heat purified *Bs*GDH.

Analytic equipment

UV-vis assays were performed on a Cary 60 or a Shimadzu UV-vis spectrophotometer UV-2401 PC, with PMMA plastic disposable cuvettes of 4 mL.

GC analyses were performed on a Shimadzu GC-2010 gas chromatograph (Shimadzu, Japan) with an AOC-20i Auto injector equipped with a flame ionization detector (FID), using helium as the carrier gas.

NMR spectra were recorded on an Agilent 400 spectrometer at 400 (^1H) and 100 (^{13}C) MHz. Chemical shifts (δ) are reported in parts per million (ppm) relative to Me_4Si (δ 0.00) using deuterated solvent (DMSO-d_6, D_2O or $CDCl_3$) as an internal standard. For NMR monitored biocatalytic conversions, a glass sealed capillary of deuterated benzene (C_6D_6) was added to the NMR tube as an internal standard. NMR data is reported as follows: br = broad, s = singlet, d = doublet, t = triplet, q = quartet, m = multiplet, ap = apparent; coupling constant(s) (*J*) in Hz; integration.

Enzyme production and purification

*Ts*OYE

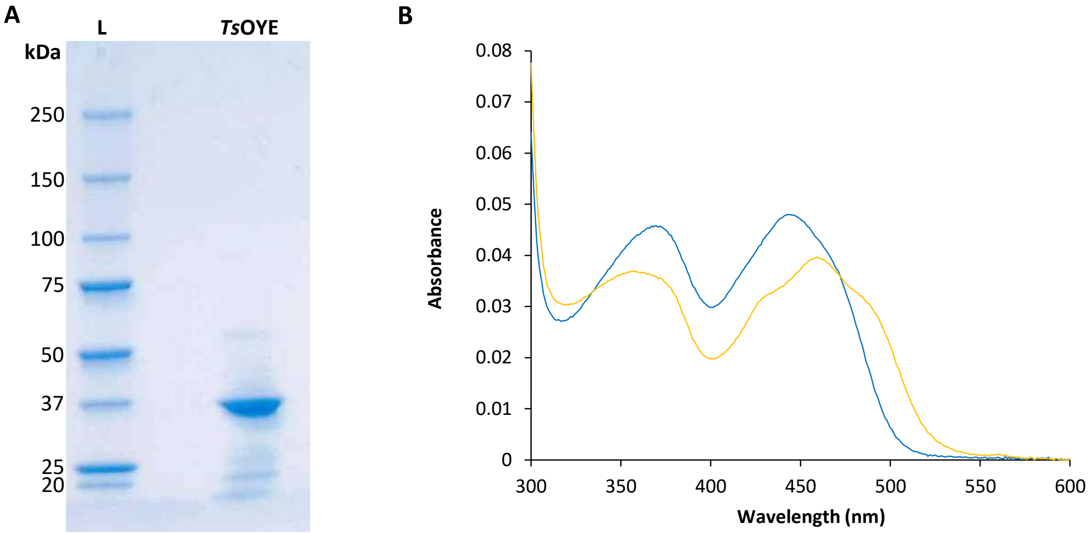

Figure S1. **A)** SDS-PAGE gel of heat-purified *Ts*OYE (36 kDa), with a Precision Plus Protein Standard ladder (L), stained with Coomassie brilliant blue. **B)** Overlay UV-vis spectra of *Ts*OYE-bound FMN with absorbance maximum at 459 nm in yellow, *Ts*OYE released free FMN with absorbance maximum at 446 nm after denaturation through the addition of 0.2% SDS, in blue.

Expression of *Ts*OYE from *Thermus scotoductus* SA-01 was performed as previously reported in *E. coli* BL21(DE3) cells (Lucigen) with a pET-22b(+)-*tsoye* plasmid (Novagen, no His-tag, ampicillin resistance) at 37 °C,[2, 6] using the Overnight Express™ Instant TB Medium (Novagen). The cells were harvested by centrifugation (10,000 rpm, 4 °C, 20 min), resuspended and washed in MOPS-NaOH buffer (20 mM, pH 7.0) and centrifuged (10,000 rpm, 4 °C, 20 min). The cell pellets were then re-suspended in MOPS-NaOH buffer (20 mM, pH 7.0) and lysed using a multi cycle cell disruptor (Constant Systems). The soluble fraction was obtained after centrifugation (10,000 rpm, 4 °C, 20 min) of the crude extract.

The recombinant *Ts*OYE was purified through heat (70 °C, 90 min), centrifuged (8,000 rpm, 4 °C, 30 min) and the supernatant was incubated with excess FMN overnight at 4 °C. The clear yellow solution was concentrated with an Amicon 30 kDa from Millipore and passed through a PD-10 desalting column from GE Healthcare with MOPS-NaOH buffer (50 mM, pH 7.0), to remove excess FMN.

Protein concentration was measured with a BCA assay using the Protein Assay Kit by Uptima with a corresponding bovine serum albumin (BSA) calibration curve. Enzyme purity was assessed by sodium dodecyl sulfate polyacrylamide electrophoresis (SDS-PAGE, **Figure S1A**). FMN concentration was measured by UV-vis spectrophotometry, using enzyme-bound FMN versus free FMN absorption measurements at 446 nm with $\varepsilon = 12{,}200\ M^{-1}cm^{-1}$ (**Figure S1B**).[7] Enzyme concentrations determined by UV-vis spectra were deemed as more accurate by measuring the concentration of FMN saturated enzyme, whereas the BCA assay measures all proteins, which could potentially include non-active *Ts*OYE without FMN or other residual proteins (<5% according to SDS-PAGE).

A 15-L fermentation was also carried out to produce *Ts*OYE, using the pET-28a(+)-*tsoye* plasmid with *N*-terminal His-tag. A total mass of 352 g wet cell pellet was obtained (23.5 g/L). An estimated 2.4 g of heat purified *Ts*OYE was produced, as described above.

Activity assays

*Ts*OYE activity was determined through monitoring NADPH consumption at 340 nm for 2 min at 30, 40 and 65 °C. Glucose oxidase (GOx) and glucose were added to consume molecular oxygen. Measurements were executed in duplicate.

General activity assay: A solution of 2 mL contained 50 mM MOPS-NaOH pH 7.0 buffer, 10 U/mL GOx, 20 mM glucose, 0.1 µM *Ts*OYE, 0.1 mM NADPH, 10 mM substrate.

Temperature stability: *Ts*OYE activity with (*R*)-carvone incubated at different temperatures for 24 h was determined through monitoring NADPH consumption at 340 nm for 1 min at 30 and 40 °C. A solution of 2 mL containing 200 mM MOPS-NaOH pH 7.0 buffer, 10 U/mL GOx, 20 mM glucose, 0.1 µM *Ts*OYE, 0.1 mM NADPH, 10 mM substrate.

Cosolvent stability: *Ts*OYE activity with (*R*)-carvone where *Ts*OYE was incubated in 1 and 20% v/v DMSO (68 h) or IAA (24 h). The activity was determined through monitoring NADPH consumption at 340 nm for 2 min at 30 and 40 °C. A solution of 2 mL containing 200 mM MOPS-NaOH pH 7.0 buffer, 10 U/mL GOx, 20 mM glucose, 0.1 µM *Ts*OYE, 0.1 mM NADPH, 10 mM substrate.

Oligomerization state

To determine the oligomerization state of *Ts*OYE in buffer and with or without DMSO cosolvent, a calibration was made for size exclusion chromatography with other known proteins (SEC, **Figure S2**). *Ts*OYE showed no difference in size with 10% v/v DMSO.

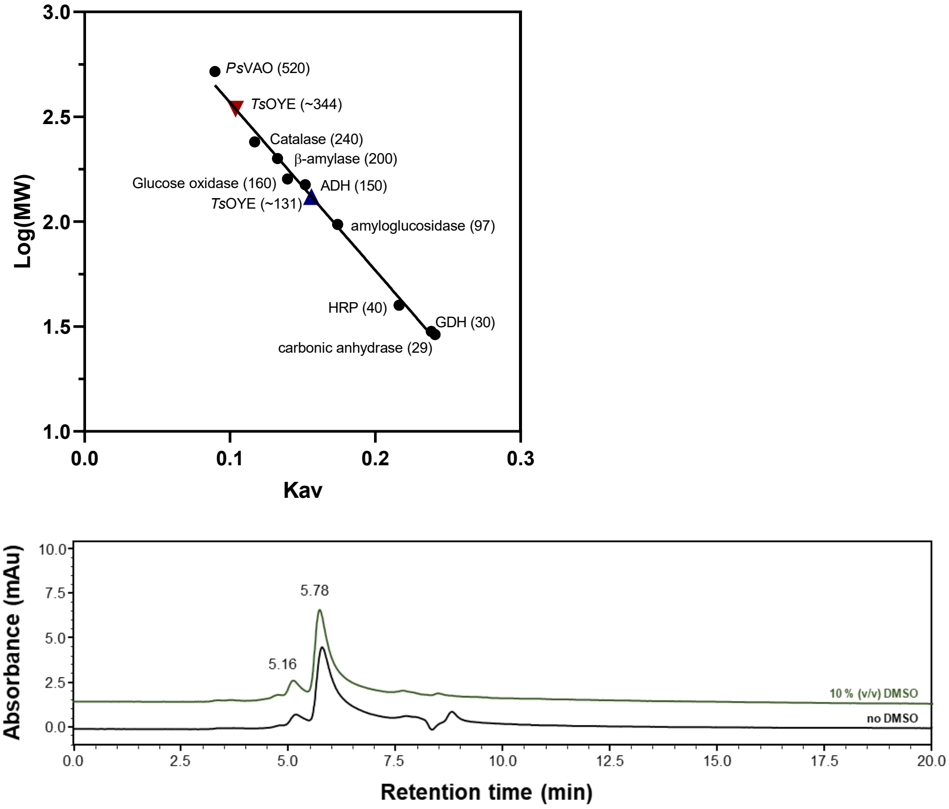

Figure S2. Size exclusion chromatography (SEC) of *Ts*OYE, **left:** calibration with other known proteins, **right:** retention time of *Ts*OYE without DMSO (black trace) and with 10% v/v DMSO (green trace).

Turnover calculations

Turnover number (TON) was calculated based on the measured conversion percentage multiplied by the concentration of the substrate added to the system (in mM) divided by the amount of enzyme added to the system (in mM).

Turnover frequency (TOF) was calculated based on TON divided by time in hours.

Cofactor stability study

Cofactor stability was evaluated by UV-vis spectrophotometry measurements of absorbance at 340 nm in 96 deep well plates. Conditions: 50-200 mM buffer, 0.4 mM cofactor, total volume = 200 µL, 30 °C, 5 h.

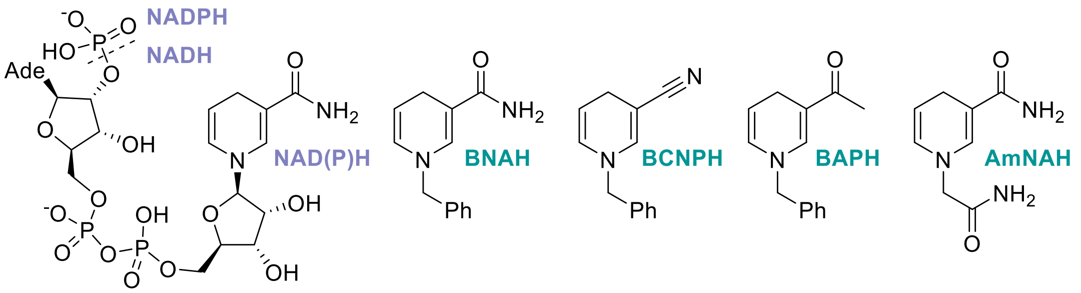

Figure S3. Chemical structures of cofactors used in stability study and/or biocatalytic reactions.

Table S1. Cofactor stability study data table. Colors in titles match the color of names of the structures (see Figure S3). The table shows a heat map where the color scale ranges from no conversion (white) to full conversion (red).

Entry	Buffer	Label	pH	[Buffer] (mM)	Rate of decay (pmol/h)					
					Natural Cofactors		Synthetic cofactors			
					NADH	NADPH	BNAH	BCNPH	BAPH	AmNAH
1	Water	MilliQ		n/a	0.2	1.5	1.6	0.8	0.6	0.5
2	NH_4Cl/ NH_4OH	NH4Cl_8	8	50	0.4	2.2	2.1	0.8	0.8	0.7
3		NH4Cl_9	9	50	0.2	0.7	1.3	0.7	0.8	0.5
4		NH4Cl_10	10	50	0.3	0.1	1.6	0.8	0.6	0.6
5		NH4Cl_9_200	9	200	0.2	0.6	1.9	1.1	0.8	0.8
6	NH_4COOH/ NH_4Cl	NH4HCO2_8	8	50	1.1	2.7	5.9	1.0	0.7	1.2
7		NH4HCO2_9	9	50	0.0	0.5	2.2	0.7	0.6	0.5
8		NH4HCO2_10	10	50	0.4	0.5	1.3	0.5	0.6	0.5
9		NH4HCO2_9_200	9	200	0.5	0.5	3.4	1.5	0.8	0.7
10		NH4HCO2_9_500	9	500	0.6	0.6	6.1	2.0	1.5	1.4
11		NH4HCO2_9_1M	9	1000	0.5	0.6	5.0	1.3	0.9	1.0
12	Na_2CO_3/ $NaHCO_3$	NaHCO3_9	9	50	0.2	0.2	1.2	0.8	0.6	0.4
13		NaHCO3_10	10	50	0.0	0.2	0.9	0.3	0.5	0.5
14		NaHCO3_11	11	50	0.2	0.2	0.9	0.3	0.5	0.8
15		NaHCO3_10_200	10	200	0.1	0.1	0.9	1.2	0.7	0.5
16	Tris-HCl	Tris_7	7	50	0.0	1.4	1.7	0.3	0.6	0.6
17		Tris_8	8	50	0.2	0.6	1.1	1.1	0.6	0.4
18		Tris_9	9	50	0.2	0.4	0.9	0.6	0.8	0.4
19		Tris_8_200	8	200	0.2	0.3	2.5	1.0	0.7	0.5
20	MOPS-NaOH	MOPS_6	6	50	3.0	13.8	16.9	2.2	1.4	2.9
21		MOPS_7	7	50	0.7	5.0	8.6	0.6	0.6	1.3
22		MOPS_8	8	50	0.2	0.7	2.3	0.5	0.7	0.7
23		MOPS_7_200	7	200	0.9	3.8	16.5	1.6	1.0	3.0
24	KPi	KPi_5	5	50	5.8	10.8	43.2	3.0	1.5	13.9
25		KPi_6	6	50	2.9	5.9	40.4	6.4	1.4	9.8
26		KPi_7	7	50	1.1	2.0	21.4	1.7	1.1	5.6
27		KPi_6_200	6	200	7.2	11.2	44.3	4.8	4.0	27.2
28	HEPES	HEPES_6.5	6.5	50	1.0	8.5	10.8	2.0	0.7	1.8
29		HEPES_7.5	7.5	50	0.4	2.3	8.2	3.3	1.2	1.1
30		HEPES_8.5	8.5	50	0.2	0.4	2.4	0.5	0.6	0.7
31		HEPES_7.5_200	7.5	200	0.6	1.6	18.1	1.2	1.0	2.7

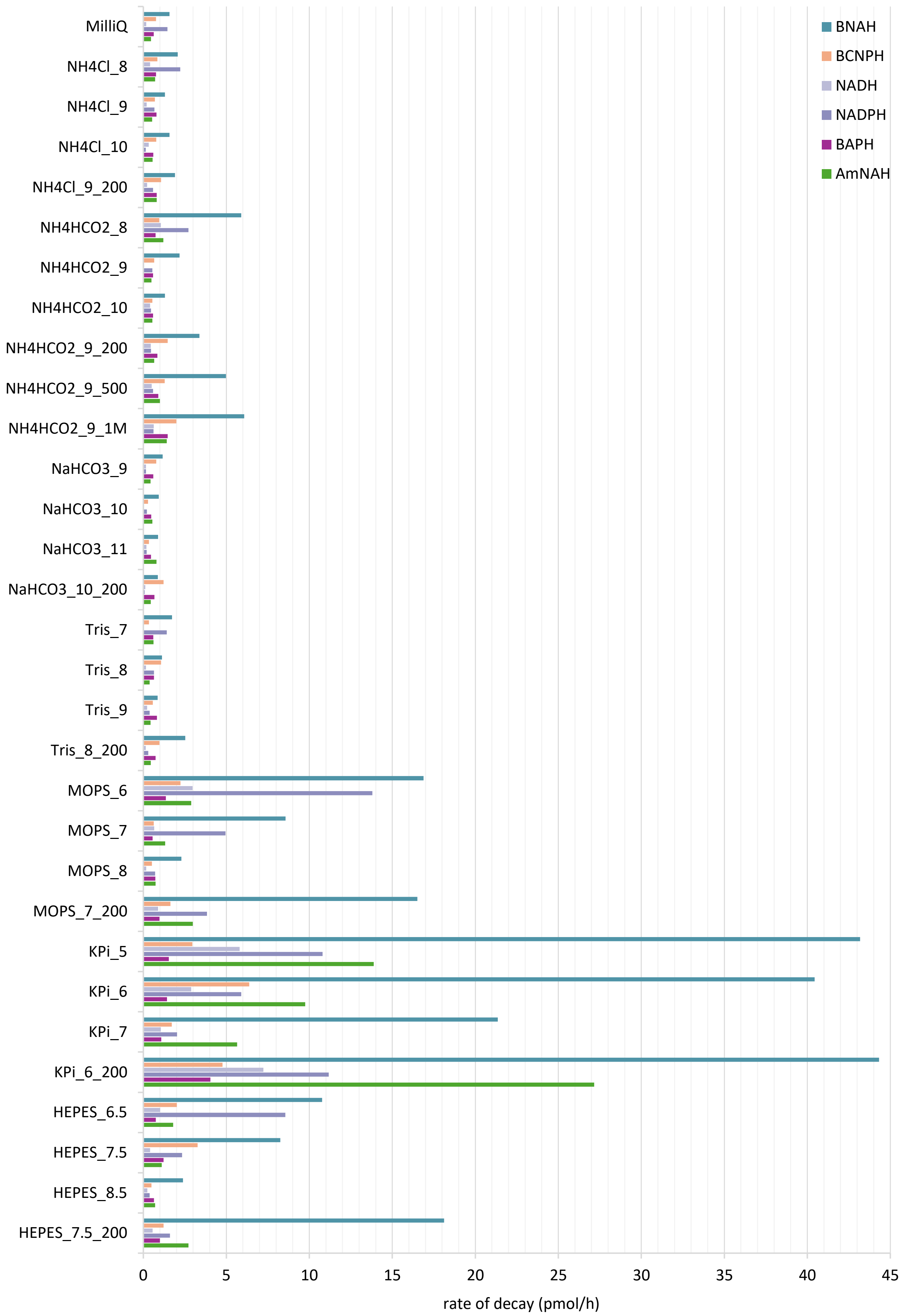

Figure S4. Overview of cofactor stability study.

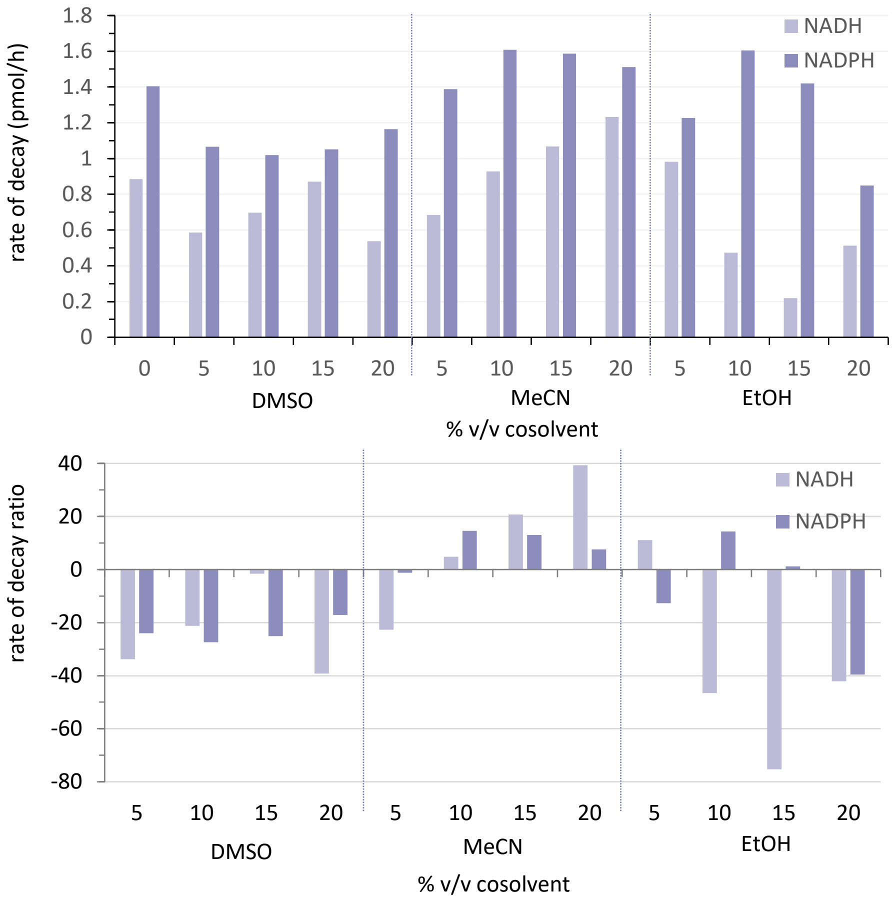

Figure S5. Stability of natural cofactors NAD(P)H in cosolvents. Cosolvent concentrations measured were 0, 5, 10, 15 and 20% v/v of DMSO, MeCN and EtOH, respectively. Ratio is relative to 0% cosolvent. Both cofactors decay slower with DMSO, and faster in >5% v/v MeCN.

Gas chromatography analyses

The GC was equipped with the chiral column Lipodex E (Macherey-Nagel), octakis-(2,6-di-*O*-pentyl-3-*O*-butyryl)-γ-cyclodextrin resin, 50 m × 0.25 mm × 0.25 µm (length, diameter, film thickness). Column flow: 2.23 mL/min, split ratio: 100, linear velocity: 38 cm/s. Calibration curves and reaction extractions had 5 mM dodecane as internal standard.

Table S2. GC oven method and retention times.

rate (°C/min) / temp (°C) / hold (min)	Compound	Ret. time (min)
0 / 80 / 2<br>5 / 110 / 5<br>5 / 130 / 5<br>20 / 220 / 1	EtOAc	2.7
	IAA	4.3
	dodecane	10.5
	(2*S*,5*S*)-dihydrocarvone	14.9
	(2*R*,5*R*)-dihydrocarvone	15.2
	(2*R*,5*S*)-dihydrocarvone	N/A
	DMSO	15.4
	(2*S*,5*R*)-dihydrocarvone	16.4
	(–)-dihydrocarveol	17.6
	(*R*)-(–)-carvone/(*S*)-(+)-carvone	18.0

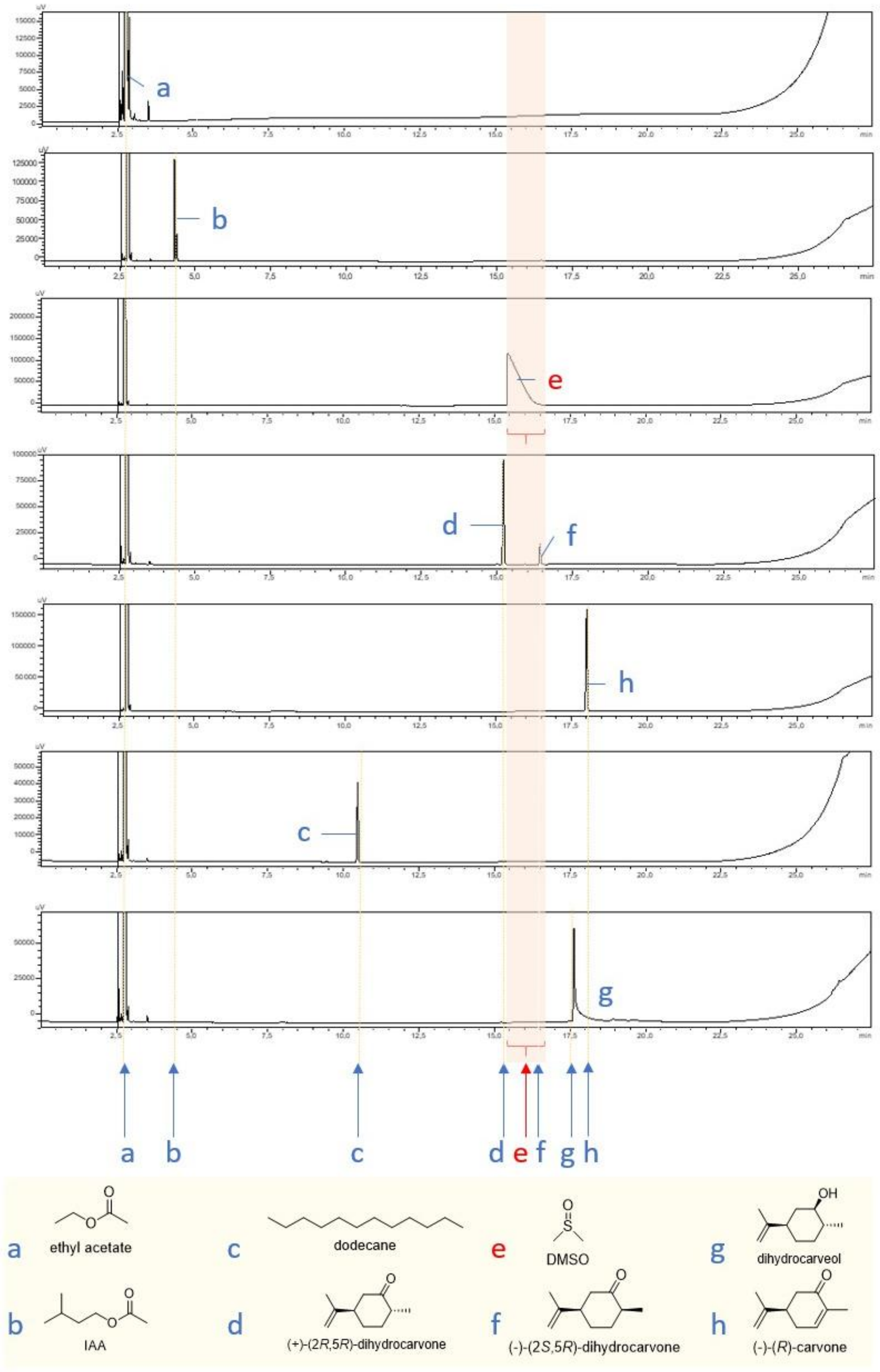

Figure S6. GC chromatograms and retention times of all compounds.

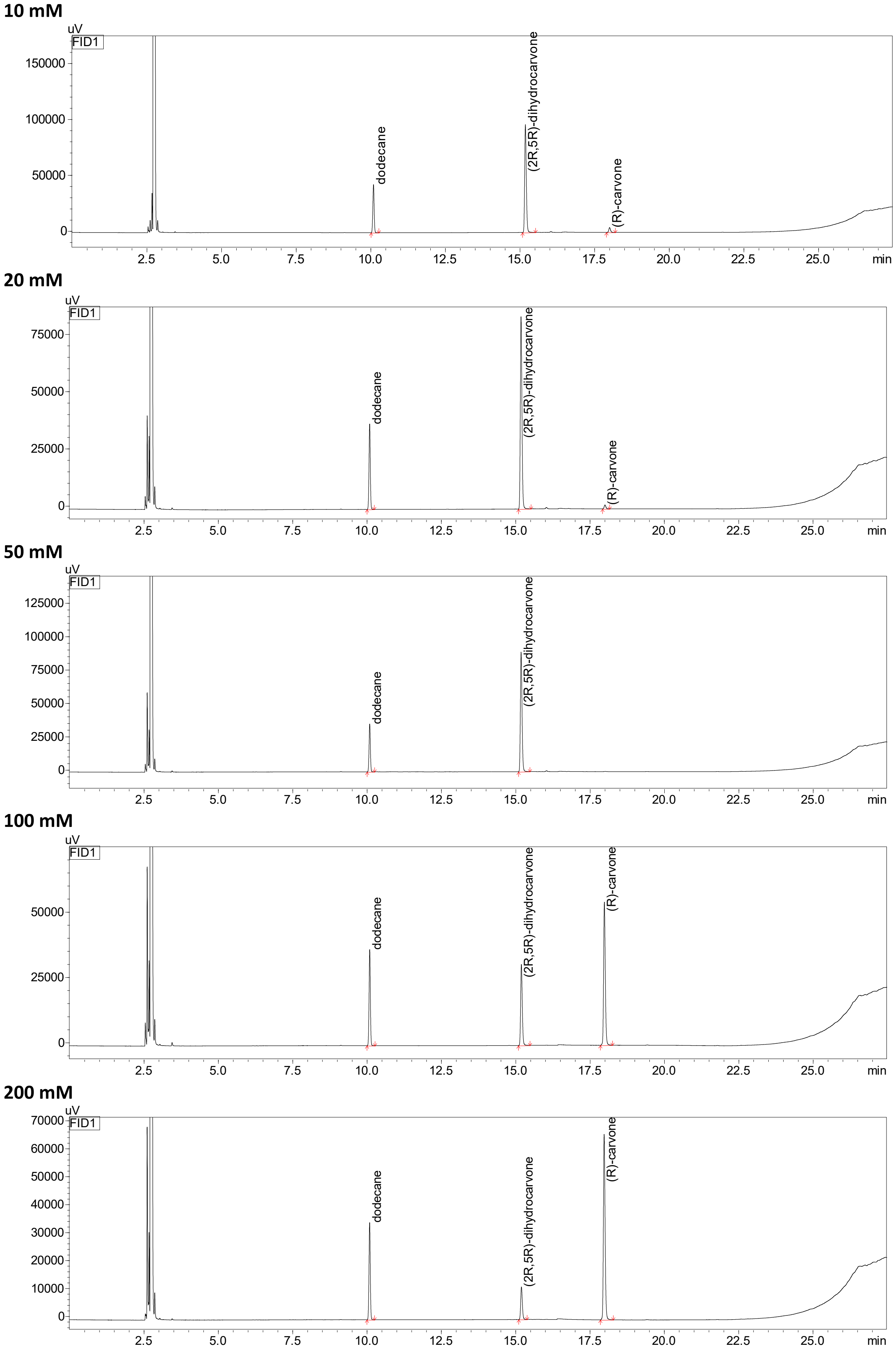

Figure S7. GC chromatograms of *Ts*OYE-catalyzed (*R*)-carvone reduction to (2*R*,5*R*)-dihydrocarvone with *Bs*GDH recycling. 10-200 mM (*R*)-carvone. See *Ts*OYE-catalyzed bioreduction reactions section in SI for general conditions.

*Ts*OYE-catalyzed bioreduction reactions

Analytical scale

The (*R*)-carvone substrate was added at various concentrations from a 1 M stock solution in DMSO, IAA or acetone. In a 2 mL Eppendorf microcentrifuge Safe-Lock tube, the reaction mixture contained the following, with the amounts specified in the figure or table captions: 50 or 200 mM MOPS-NaOH pH 7.0 or 200 mM KPi pH 7.0 buffer, the cofactor system (see below), (*R*)-carvone, the *Ts*OYE enzyme, in an Eppendorf ThermoMixer (at 30 °C, 700 rpm) for the desired time, to produce (2*R*,5*R*)-dihydrocarvone (major product) and (2*S*,5*R*)-dihydrocarvone (minor product). The reaction was quenched by extraction with 0.5 mL EtOAc, vortexed and centrifuged (13,000 rpm, 2 min). The organic phase was diluted with EtOAc to 10 mM if the substrate was >10 mM, and dried with $MgSO_4$ (13,000 rpm, 1 min), decanted to a clean GC vial then measured on a gas chromatography (GC). All data points are averages of duplicates.

With GDH-catalyzed NADPH cofactor recycling

For the recycling system, 0.1 or 0.2 mM $NADP^+$ was added along with the corresponding amount 100, 200 or 220 mM of D-glucose monohydrate, and 10 mg/mL GDH (from Evocatal) or 3-9 U/mL *Bs*GDH.

With synthetic cofactors

Stoichiometric amounts with 10% excess of cofactor BNAH or AmNAH were weighed in the vial as a solid powder.

Preparative scale

Either BNAH or the cofactor recycling system along with MOPS buffer were introduced in a 250 mL 3-neck round-bottom flask, and substrate was added. The reaction flask was placed in an oil bath at 30 °C. Top-stirring, nitrogen flow, pH-meter and pH-adjustment system (Dosimat, 1 M HCl solution for BNAH, 1 M NaOH solution for NADPH recycling, rate of volume addition (dV/dt) = 6, Metrohm 5 mL) were set-up. The *Ts*OYE enzyme was added, and the reaction was carried out in the dark with aluminium foil. For the reaction with BNAH, initially the pH increased quickly, and the volume addition rate was adapted.

To compare the catalytic efficiency and the productivity of *Ts*OYE for small and large scale bioreduction, the turnover frequency (TOF), turnover number (TON) and productivity number (PN) were calculated (**Table S3**).

Table S3. TOF, TON and PN values for small and large-scale bioreduction. [*Ts*OYE]= 0.05 g/L (1.4 µM), reaction time: 5 h, MOPS concentration: 42 g/L (200 mM pH 7.0).

V_{tot} (mL)	Substrate (g)	Substrate (mmol)	[Enzyme] (µM)	Conv. (%)	TOF (s^{-1})	TON	PN (mol/g/h)
1	0.10	0.6	1.4	80	19.0	343000	1.92
100	10.4	60	14	43	10.2	184000	1.03

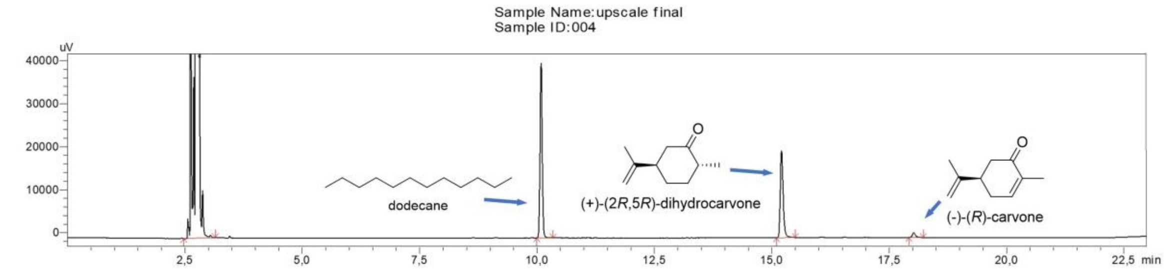

Figure S8. GC chromatogram of product from 100 mL scale up. The chromatogram showed the enantiopure (2*R*,5*R*)-dihydrocarvone product from scale-up of 100 mL with 1 M (*R*)-carvone, 8 µM *Ts*OYE, 1 mM $NADP^+$, 3 U/mL *Bs*GDH, 1 M glucose in 200 MOPS-NaOH buffer pH 7.0. Reaction was stopped after 102 h.

Supplementary figures

Table S4. Comparison of >2 g/L scale ERED-catalyzed asymmetric alkene reductions

	Substrate	(g)	(g/L)	ERED	Conv./ product yield		(%)	*ee R* (%)	TON	Ref.
**8a**	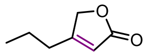	0.13	2.5	HYE2_T67F_ S140Y_E248D	**8b**	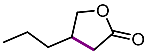	81[a]	99	705	8
**9a**	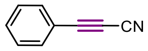	0.05	5	OYE3	**(*E*)-9b**	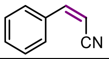	97	98:2[f]	5,600[c]	9

[a] isolated yield.

Table S5. Influence of pH and temperature on the *Ts*OYE specific activity[a]

Substrate	pH	Temp. (°C)	Spec. act. (U/mg)
cyclohexenone	7.0	20	12.0
cyclohexenone	8.0	20	12.2
cyclohexenone	8.5	20	6.5[c]
cyclohexenone	9.0	20	7.3
(*R*)-carvone	7.0	30	5.7
(*R*)-carvone	7.0	40	9.5
(*R*)-carvone	7.0	65	18.1
(*R*)-carvone	8.5	20	4.4[c]
(*S*)-carvone	8.5	20	2.6[c]

[a] Conditions: buffer, 0.2 mM NADPH, 10 mM substrate with 1% v/v DMSO, *Ts*OYE; [b] buffers: 50 mM MOPS-NaOH pH 7; 50 mM Tris-HCl pH 8; 200 mM Tris-HCl pH 8.5; 50 mM Na_2CO_3 pH 9. [c] Lower specific activity could also be due to higher 200 mM buffer concentration.

10-200 mM (*R*)-carvone

With cosolvents

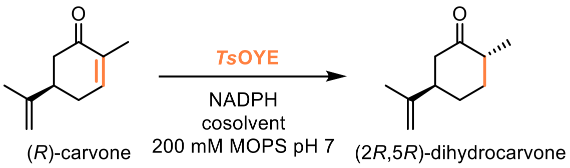

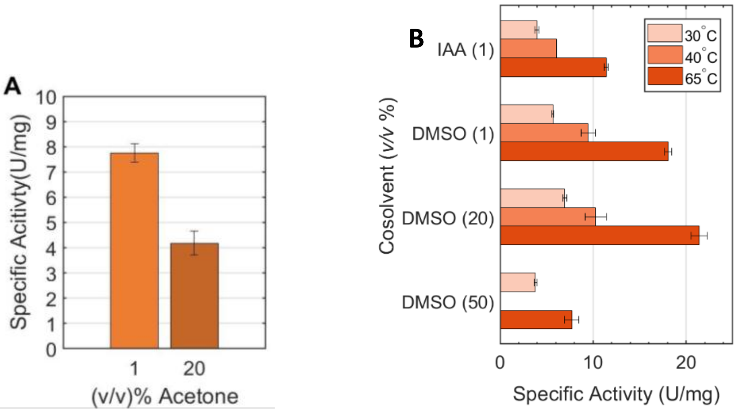

Figure S9. *Ts*OYE specific activity with cosolvents acetone, IAA and DMSO. Conditions: general assay conditions with the buffer 200 mM MOPS-NaOH pH 7. **A)** With 1 or 20% v/v acetone at 30 °C (with 0.2 mM NADPH). **B)** With 1% v/v IAA, 1, 20 and 50% v/v DMSO at 30, 40 or 65 °C. No data point for 50% v/v DMSO at 40 °C. Data points are an average of duplicate measurements of slopes from NADPH depletion with UV-vis at 340 nm.

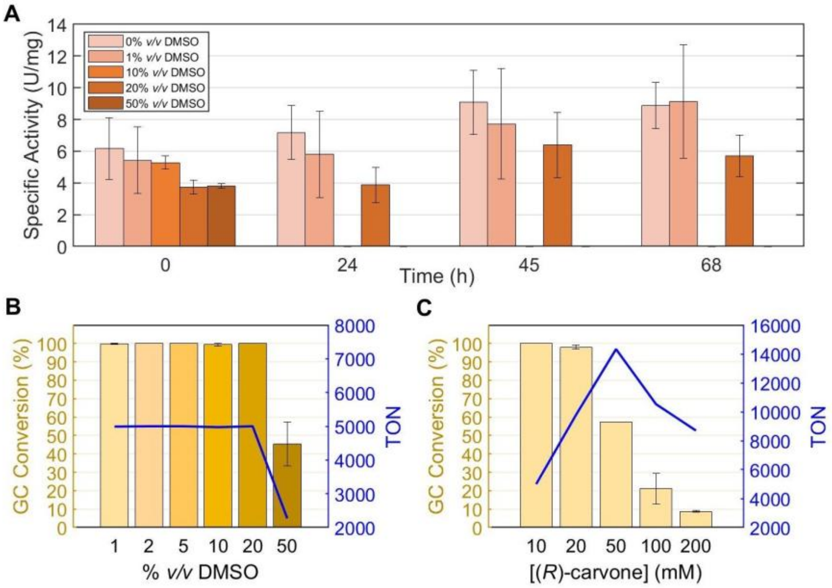

Figure S10. The influence of DMSO as cosolvent on *Ts*OYE activity and conversion.

A) Activity of *Ts*OYE with varying % v/v DMSO. Conditions: General assay conditions with 200 mM MOPS-NaOH buffer pH 7.0 at 30 °C. Measured at 340 nm, average of triplicates.

B) Bioconversion of 10 mM (*R*)-carvone in 1-50% v/v DMSO; **C)** Bioconversion of 10-200 mM (*R*)-carvone with 1% v/v DMSO. Conditions of **B)** and **C)**: 50 mM MOPS-NaOH pH 7.0, 350 U/mL GDH (Evocatal), 100 mM glucose (limiting factor), 0.1 mM $NADP^+$, 2 µM *Ts*OYE, 1 h reaction, 30 °C, 900 rpm, 1 mL volume. Data points are an average of duplicate samples analyzed by GC.

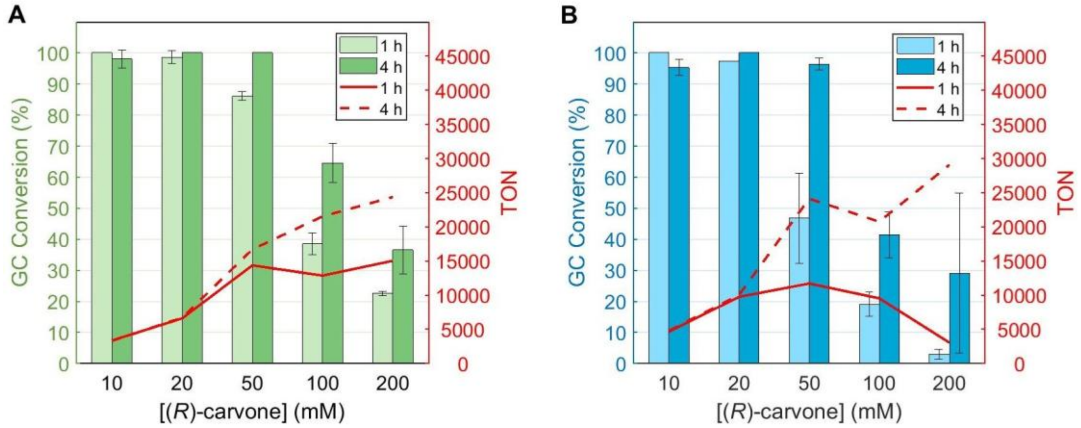

Figure S11. The effect of enzyme and substrate concentration. **A)** 3 µM *Ts*OYE. **B)** 2 µM *Ts*OYE. Conditions: 50 mM MOPS-NaOH pH 7.0 containing, 100 mM glucose, 350 U/mL GDH (Evocatal), 0.1 mM $NADP^+$, 1, 2, 4, 8 and 17% v/v DMSO for the 10, 20, 50, 100 and 200 mM samples, respectively, 1 mL total volume, 30 °C, 900 rpm, for 1 and 4 h. Data points are an average of duplicate samples analyzed by GC.

200-800 mM (*R*)-carvone

With synthetic cofactors BNAH and AmNAH

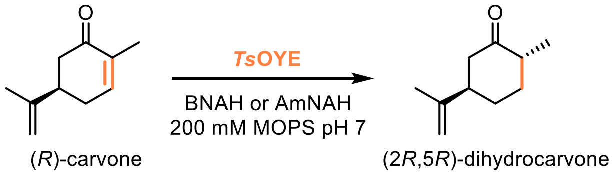

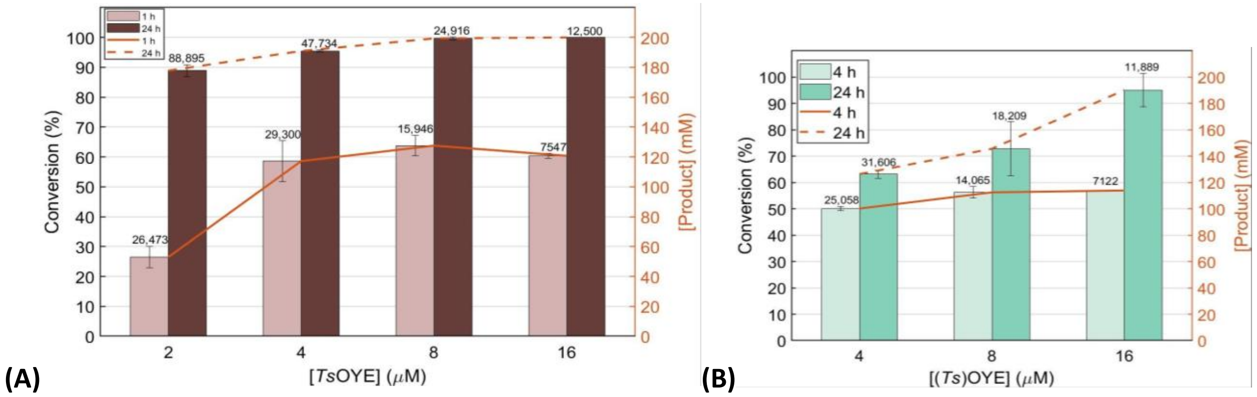

Figure S12. 200 mM (*R*)-carvone with varying *Ts*OYE concentrations and **(A)** BNAH or **(B)** AmNAH. Conditions: 220 mM BNAH or AmNAH added as a solid powder, 200 mM MOPS-NaOH pH 7.0, 2, 4, 8, or 16 µM *Ts*OYE, 200 mM (*R*)-carvone from a DMSO stock (17% v/v DMSO final), 1 mL total volume, 30 °C, 900 rpm, 1 or 4 h and 24 h. Data points are an average of duplicate samples analyzed by GC.

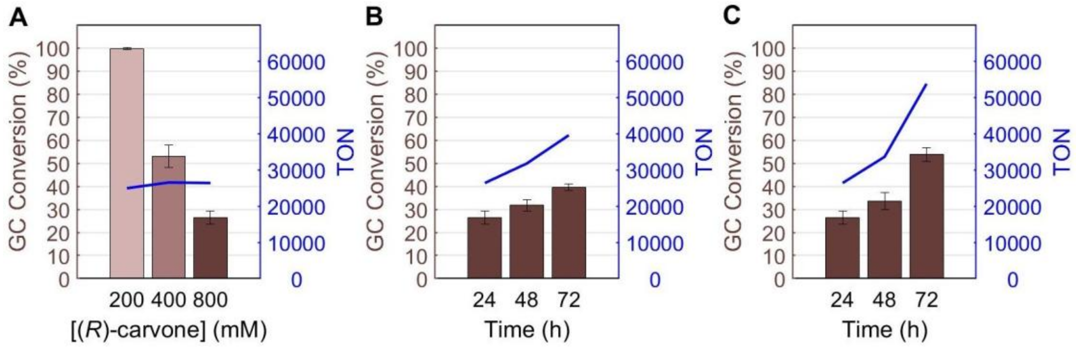

Figure S13. The effect of increasing substrate concentration and reaction time with BNAH. **A)** Increase of (*R*)-carvone concentration 200, 400 and 800 mM in a 24 h reaction time using 220, 440 and 880 mM BNAH, respectively. **B)** increase of reaction time for 800 mM (*R*)-carvone using 880 mM BNAH. **C)** same as **B)**, but BNAH was added in increments. General reaction conditions: 200 mM MOPS-NaOH pH 7.0, 8 µM *Ts*OYE, substrate with 20% v/v DMSO, cofactor, 1 mL volume, 30 °C, 900 rpm. Data points are an average of duplicate samples analyzed by GC

With GDH cofactor recycling

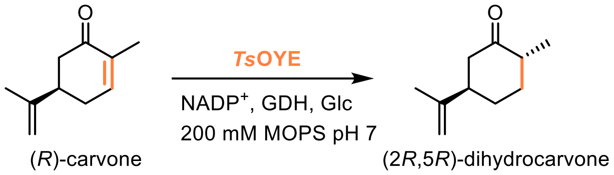

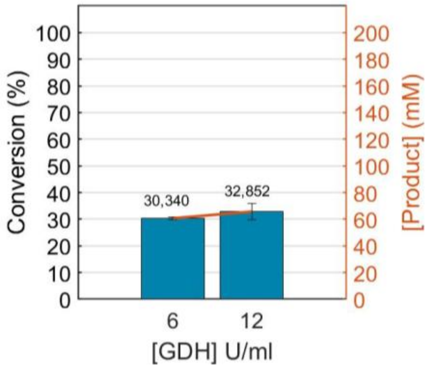

Figure S14. Effect of increased *Bs*GDH concentration. Conditions: 200 mM MOPS-NaOH pH 7.0, 200 mM glucose, 0.1 mM NADP+, 6 or 12 U/mL *Bs*GDH, 2 µM *Ts*OYE, 200 mM (*R*)-carvone added from a DMSO stock, 1 mL total volume containing 17% v/v DMSO, 30 °C, 900 rpm, 24 h reaction. TON are written above the bars. Data points are an average of duplicate samples analyzed by GC.

800 mM- 1 M carvone

10 mL volume

Table S6. Scale up 1 M substrate in 10 mL volume in absence of DMSO.[a]

[*Ts*OYE] µM	Cofactor	Initial[b] TOF (conv. %)	Final[c] TOF (conv. %)
1	$NADP^+$	266 (0.7)	26 (0.7)
8	$NADP^+$	326 (6)	126 (26)
1	BNAH	9929 (25)	1070 (28)

[a] Conditions: 1 M (*R*)-carvone, 30 °C, 600 rpm, 200 mM MOPS-NaOH pH 7.0 in 10 mL volume. Cofactor $NADP^+$ (1 mM) with *Bs*GDH (3 U/mL) and glucose (1.1 M). Synthetic cofactor BNAH (1.1 M) was added in increments of 200 mM per day. GC conversions derived from single aliquots (10 µL).
[b] Initial turnover frequency (TOF) measured at 22.5 h for $NADP^+$ and 25 h for BNAH
[c] Final TOF was measured at 206 h.

50-100 mL volume

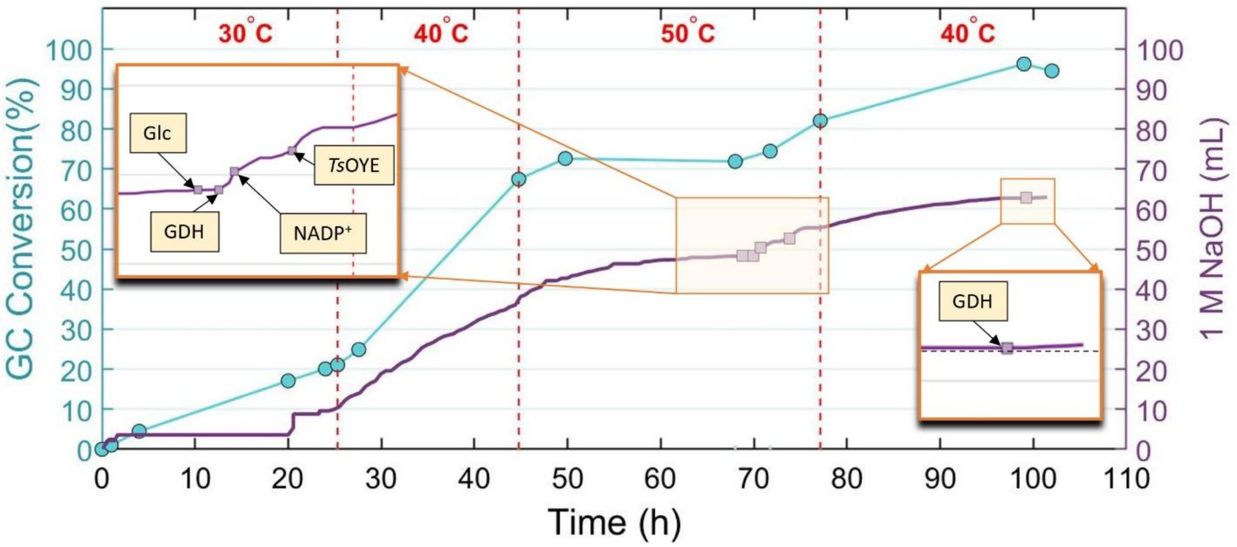

Figure S15. Scale-up of *Ts*OYE-catalyzed reduction of 800 mM (*R*)-carvone in 100 mL volume. **Blue circle markers** represent samples taken and measured by GC. **Purple line** indicates the amount of base added (1 M NaOH) to maintain pH 7.0. **Purple square markers** represent time points when components were added. The *de* for all data points was >99.9%. Conditions: 100 mL total volume, 200 mM MOPS-NaOH pH 7, 880 mM glucose, 3 U/mL *Bs*GDH, 1 mM $NADP^+$, 8 µM *Ts*OYE, 800 mM (*R*)-carvone from 30 to 50 °C, top stirrer. Additions: glucose (2.5 mmol at 68.8 h), *Bs*GDH (12 U/mL at 69.9 h), $NADP^+$ (0.2 mM at 70.8 h), *Ts*OYE (8 µM at 74 h), *Bs*GDH (18 U/mL at 99.3 h). 102 h, 94% conversion 94,406 TON.

A scale-up with 800 mM (*R*)-carvone for which a maximum of 100 g/L of product could be achieved (**Figure S15**). No DMSO cosolvent was used to aid in extraction of pure product. The temperature started at 30 °C and was to be increased to 40, then 50 °C, to see effects of temperature at a higher volume and concentration. A pH meter with base injections (2 M NaOH) was used to maintain the pH at 7.0, to avoid acidification that could denature the enzymes. The volume was set at a manageable 100 mL with a top-stirring mechanism, starting at 120 rpm, then slowed to 60 rpm as the reaction was emulsifying and the pH meter readings were sporadic and inaccurate. The rate of conversion increased with the increase of temperature for the second 24 h with a slope of 2.5, reaching 70% conversion. The temperature was then set to 50 °C, however the rate of conversion dropped to a slope of 1.0 for the first 5 h then to a slope of 0.09 overnight. The high temperature may have caused inactivation of one or both enzyme(s). Four components (glucose, NADP, *Bs*GDH and *Ts*OYE) were added in 40 min intervals and monitored for a change of base influx. At 68.6 h, 1 mL of a 25 M solution of glucose was added. This resulted in no change of activity. At 69 h, as 3 U/mL *Bs*GDH was added, there was a notable slope increase. Next $NADP^+$ was added at 69.4 h. The slope curve remained stable. Finally, *Ts*OYE was added, which resulted in an increasing slope. To possibly speed conversion further, more *Bs*GDH was added (3 U/mL).

The combined addition of these two enzymes increased the slope to 1.4 during the next 5.5 h. To ensure the conversion would continue and avoid further enzyme inactivation, the temperature was lowered to 40 °C after 78 h. After 99.3 h a final injection of *Bs*GDH was added (18 U/mL). The final 24 h resulted in a conversion increase of 8% and an average slope of 0.7. The total reaction ran for 102 h and gave 94% conversion to the enantiomeric pure product according to GC analysis. The ^{1}H NMR spectrum showed a purity of 83 ± 6%. The theoretical yield of product (2*R*,5*R*)-dihydrocarvone was 12.18 g with an estimated 0.57 g lost due to sampling. The adjusted net theoretical yield (subtracting out lost product) was 11.61 g and a final yield of 9.29 g (80%) where a total loss of 5% yield was due to sampling.

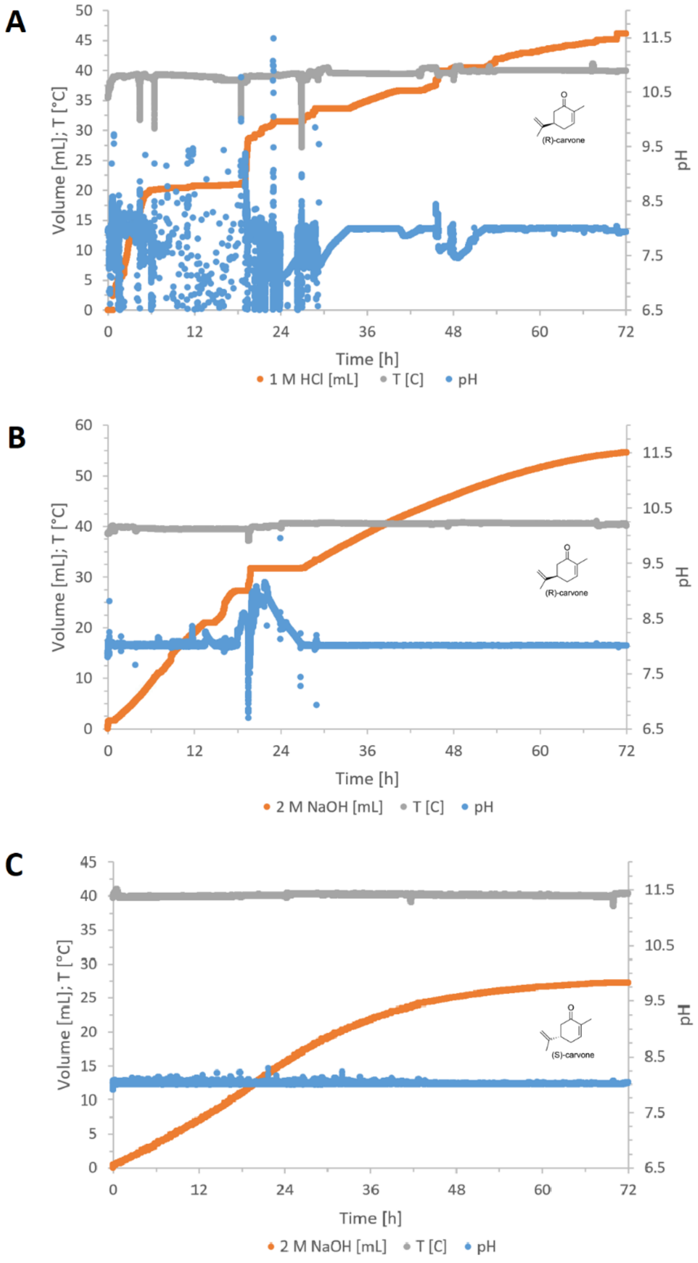

Figure S16. *Ts*OYE scale-up reactions with 1 M (*R*)- or (*S*)-carvone substrate with either BNAH or NADPH recycling, in 50 or 100 mL volume.

A) 1 M (*R*)-carvone with synthetic cofactor BNAH in 50 mL reaction volume. 85% conversion was observed by GC. Total isolated product was 6.5 g of (2*R*,5*R*)-dihydrocarvone. TON based on conversion: 106,120. Reaction conditions: 40 °C, 1.3 M (14.2 g) BNAH added in increments, 3 U/mL *Bs*GDH, 8 µM *Ts*OYE, 200 mM Tris-HCl pH 8. pH control with a dosimeter adding a solution of 1 M HCl. The scattered pH data points were due to poor stirring of the reaction mixture in the first day of the reaction.

B) 1 M (*R*)-carvone with NADPH recycling in 100 mL reaction volume. 98% conversion was observed by GC. Total isolated product was 11.8 g of (2*R*,5*R*)-dihydrocarvone. TON based on conversion: 115,765.

C) 1 M (*S*)-carvone with NADPH recycling in 50 mL. 98% conversion was observed. Total isolated product was 6.9 g (90%) of (2*R*,5*S*)-dihydrocarvone. TON based on conversion: 123,000.

B) and **C)** Reaction conditions: 200 mM Tris-HCl pH 8, 1.1 M glucose, 3 U/mL *Bs*GDH, 1 mM NADP^{+}, 8 µM *Ts*OYE, 40 °C. pH control with a dosimeter adding a solution of 2 M NaOH.

Table S7. The TON and yield summary of the *Ts*OYE scale-up reactions.

Substrate (1 M)	Cofactor	Volume (mL)	Conv. (%)	Yield (g)	TON
(*R*)-carvone	BNAH	50	85	6.5	106,120
(*R*)-carvone	NADPH recycling	100	98	11.8	115,765
(*S*)-carvone	NADPH recycling	50	98	6.9	123,000

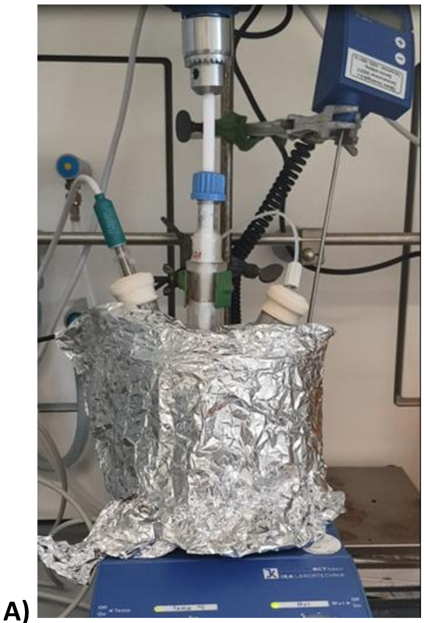

A)

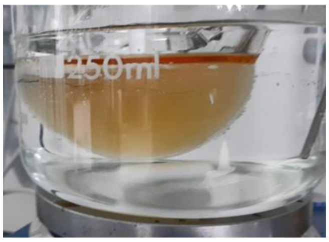

B)

Figure 17. Experimental set up. **A)** in a 250 mL flask in a water bath that is stirred and with temperature control, a pH probe and NaOH addition tube shown entering the two sides of the flask, and the center stirring bar that was gently stirred, covered in tinfoil to avoid light. **B)** a noticeable dark yellow/orange organic phase layer on top of the aqueous phase; note that no cosolvent was added.

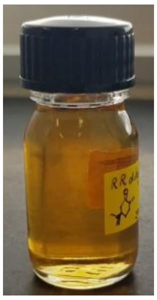

Figure S18. Final isolated (2*R*,5*R*)-dihydrocarvone product with >99% *de* obtained from the 100 mL (150 g/L) scale-up.

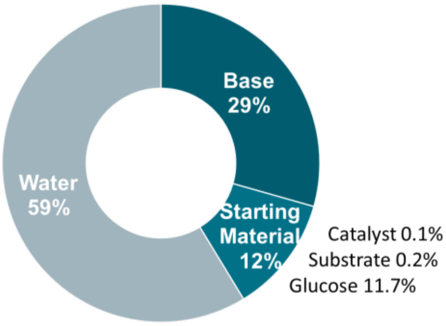

Figure S19. Environmental E-factor calculation breakdown, E-factor = 11.6. The calculation does not include the extraction solvent (diethyl ether).

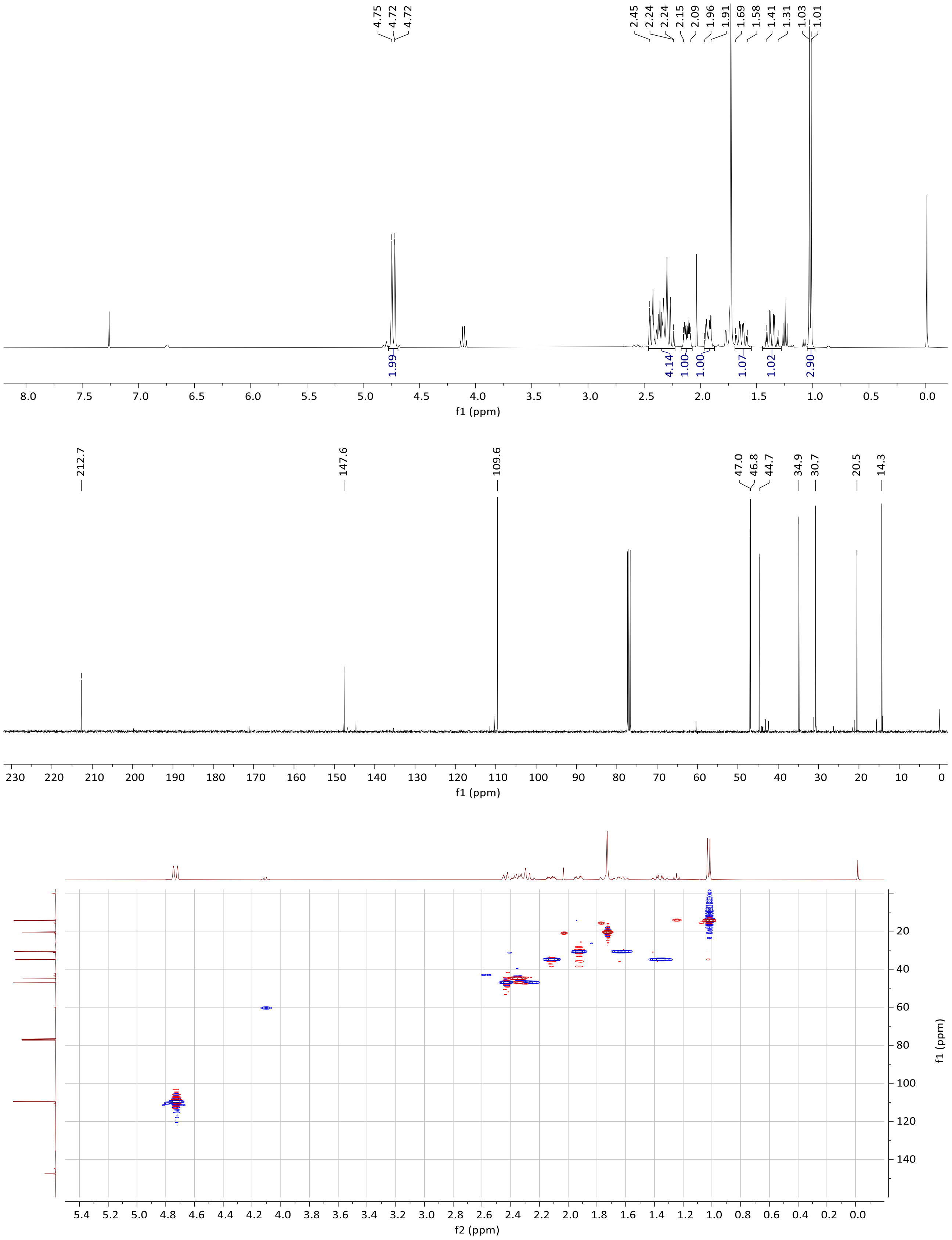

Figure S20. ^{1}H, ^{13}C and HSQC NMR spectra (in $CDCl_3$ with TMS) of dihydrocarvone product obtained from the 100 mL (150 g/L) scale-up. Trace amounts of EtOAc were observed.

NMR *in situ* monitoring

Calibration of each component was performed. The start time was set as 0 when the enzyme was added and the NMR tube inserted in the probe. Shimming and tuning gave an average first delay time of 4 min, and measurements were recorded every 5 min thereafter. The reaction was followed over time through integration of determined pre-calibrated peaks. All spectra were corrected for phase and baseline.

The NMR *in situ* monitoring was initially tried using different deuterated solvents, $CDCl_3$, D_2O, and benzene-d_6 (in a capillary). The best results to monitor the decrease or increase of peaks were with benzene-d_6 in a closed capillary inserted into the reaction mixture, such that the deuterated benzene was separated and could not interfere with the enzymatic reaction in aqueous media, and the peak was far enough from the other peaks of interest.

A benzene-d_6 capillary was obtained commercially (Sigma-Aldrich) and was used as internal standard in NMR water suppression experiments using the PRESAT pulse sequence. The benzene-d_6 signal (s, 7.16 ppm) was used as reference and its peak integral was normalized. The chemical shifts of (*R*)-carvone δ: 6.75 (ddt, *J* = 5.7, 2.8, 1.3 Hz, 1H), 4.82 – 4.77 (m, 1H), 4.75 (s, 1H), 2.74 – 2.62 (m, 1H), 2.62 – 2.53 (m, 1H), 2.50 – 2.21 (m, 3H), 1.81 – 1.72 (m, 6H) and dihydrocarvone δ: δ 4.75 (t, *J* = 1.5 Hz, 1H), 4.71 (d, *J* = 15.3 Hz, 1H), 2.49 – 2.39 (m, 1H), 2.39 – 2.22 (m, 3H), 2.12 (ddt, *J* = 12.8, 6.5, 3.4 Hz, 1H), 1.99 – 1.77 (m, 2H), 1.73 (d, *J* = 1.7 Hz, 3H), 1.68 – 1.56 (m, 1H), 1.37 (qd, *J* = 13.1, 3.5 Hz, 1H), 1.06 (dd, *J* = 22.2, 6.8 Hz, 3H), were used to follow the conversion over time.

Commercially available 3.0 mm capillary tubes (purchased from Sigma-Aldrich) containing deuterated benzene benzene-d_6 (C_6D_6) were sealed and used as internal standard in NMR experiments, used for shimming and locking, with a chemical shift at 7.16 ppm.

Water suppression programs such as the WATERGATE[10] (WATER suppression by GrAdient Tailored Excitation) with ES[11] (Excitation Sculpting) or presat PURGE[12] (Pre-saturation Utilizing Relaxation Gradients and Echoes) pulse sequences.

General conditions of the *in situ* NMR experiments: 50 mM or 200 mM (*R*)-carvone or 50 mM cyclohexenone; WATERGATE_ES or presat PURGE; 16 or 64 scans; spin (20 Hz) or no spin; 4 or 17% v/v DMSO; 55, 200 or 210 mM glucose; 0.1 or 0.2 mM $NADP^+$, 30 °C, 200 mM KPi buffer pH 7.0, 3 U/mL *Bs*GDH, 2 µM *Ts*OYE, in 0.3, 0.5 or 1 mL volume; 5 or 15 min intervals.

Reactions were initiated by adding a volume of substrate stock to the enzyme solution. The solution was transferred to a clean dry NMR tube containing the calibrated capillary followed by immediate degassing of the headspace. A series of spectra were then acquired at the desired time intervals.

(*R*)-Carvone reduction

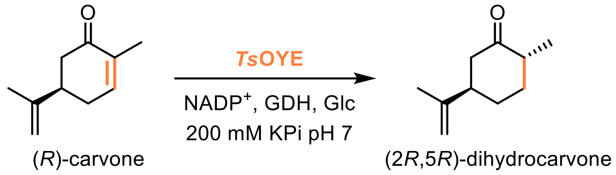

^{1}H NMR spectra were run for each component of the reaction; substrate, product, cosolvent, glucose and buffer. The spectra were analyzed to find which peaks did not overlap (**Figure S21** and **Figure S22**). The peak at 6.9 ppm (substrate) and range of peaks between 0.9 and 1.5 ppm (product) were selected to monitor the reaction. Two buffers, KPi and MOPS, were also compared. KPi showed two peaks (0.2 and 4.7 ppm), whereas MOPS showed several chemical shifts (0.2, 2, 3, 4 and 5 ppm).

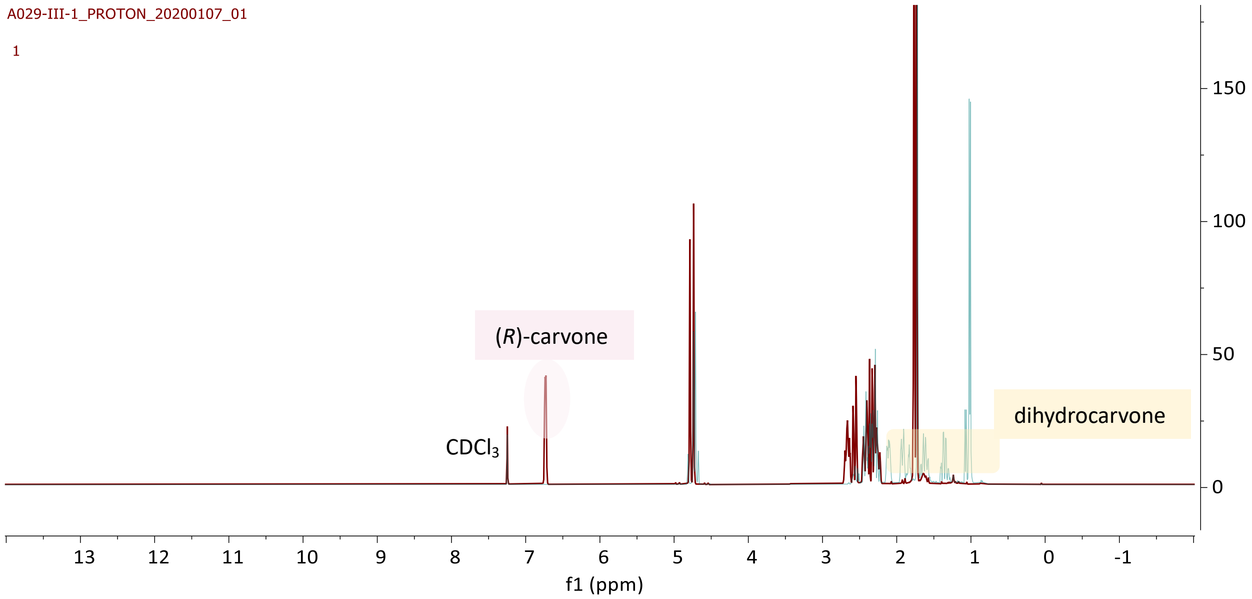

Figure S21. **^{1}H NMR spectrum overlay of (*R*)-carvone substrate and dihydrocarvone product in $CDCl_3$.** Around 6.75-6.9 ppm the (*R*)-carvone peak does not overlap with the dihydrocarvone product. Product peaks from 1.0 to 1.5 ppm do not overlap with substrate. These peaks are far from the water peak around 4.79 ppm and were used to monitor the reaction.

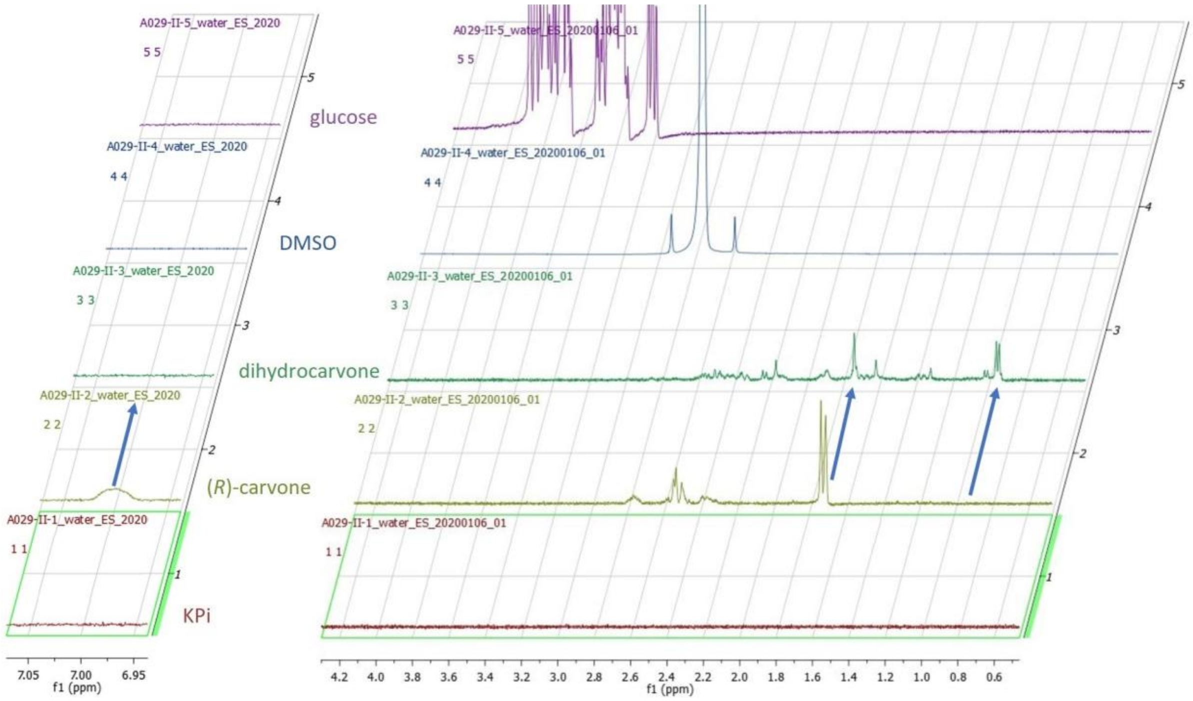

Figure S22. ^{1}H NMR spectra of each reaction component.

A calibration curve was created (**Figure S23**) using a low range of substrate concentrations (1 to 50 mM), to determine concentration as well as detection limit on the ^{1}H NMR. All peaks were visible with integrals that were quantifiable for ≥5 mM with signal to noise ratios (SNR) above 10:1. The calibration points, when connected with a shape preserving line, shows the flattening of a curve. The linearity starts to degrade over 10 mM of concentration. This concentration is close to the water solubility of the substrate (*ca.* 9 mM). For the ^{1}H NMR spectra calibration line for substrate concentration determination, an experiment was set up with six different substrate concentrations (1, 5, 10, 20, 30 and 50 mM) including an internal standard (deuterated benzene in a closed capillary placed inside the NMR tube). There were three proton spectra measured at each concentration, relating to different scans (64, 16 and 8).

From the integral area of the deuterated benzene peak (7.1 ppm) and (*R*)-carvone peak (6.9 ppm), a response factor (RF) was derived using the equation "(normalized factor) = (area substrate)/(area internal standard)", where substrate is (*R*)-carvone, and the internal standard is deuterated benzene. The normalized areas were plotted against the concentrations with a linear trend line (**Figure S23**). The R^2 value for 16 scans was 0.7255. The detection limit (DL) and quantification limit (QL) can be measured by signal to noise ratio, where a DL is generally accepted to be 3:1 and QL to be 10:1.

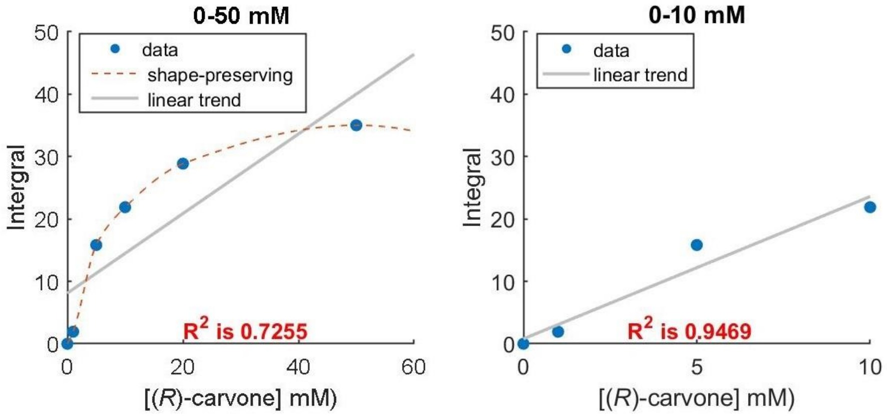

Figure S23. **^{1}H NMR spectra calibration line.** 16 scans, 5 data points from various concentrations of (*R*)-carvone normalized with integral area of deuterated benzene peak. Left: all data points with linear trend line and shape preserving line. Right: Lower concentrations ≤10 mM as water solubility for (*R*)-carvone is ~9 mM.

Calibration line for (*R*)-carvone: a ^{1}H NMR calibration line was created with 16 scans with an R^2 value of 0.7255 (**Figure S23**). Signal to noise ratios were calculated using Agilent VNMRJ software for calibration points measured under 64, 16 and 8 scans (**Table S8**).

Table S8. Signal to Noise ratios of calibration points NMR.

	Signal to noise ratio (SNR)		
	Number of scans		
(*R*)-carvone (mM)	8	16	64
1	1.8	2.2	5.1
5	10.4	14.1	39.6
10	12.6	16.8	37.7
20	18.5	27.5	43.5
30	18.9	26.3	56.2
50	23.4	35.5	64.9

Three reactions were carried out. The first reaction had 200 mM (*R*)-carvone in KPi buffer pH 7.0, as MOPS buffer had multiple peaks on the spectra compared to KPi. The volume in the NMR tube initially was too high (1 mL) such that the activity of conversion was outside the NMR signal window, and it was suspected that diffusion was measured instead of conversion (**Figure S24**). The reaction ran for 14 h (**Figure S25**). The NMR reaction solution was extracted and analyzed by GC, showing only 43% conversion.

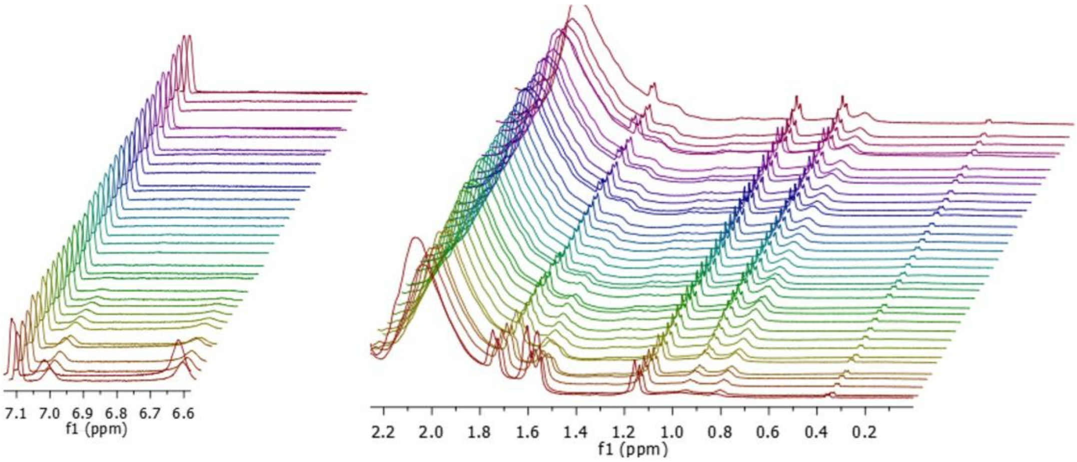

Figure S24. ^{1}H NMR experiment with 200 mM substrate. Conditions: 200 mM KPi-HCl buffer pH 7.0, 210 mM glucose, 3 U/mL *Bs*GDH, 0.1 mM NADP$^+$, 200 mM (*R*)-carvone added with 17% v/v DMSO, 2 µM *Ts*OYE, 30 °C, solvent locking with C_6D_6 in a closed capillary; 15 min measurement intervals over 15 h with water_ES pulse program; 64 scans.

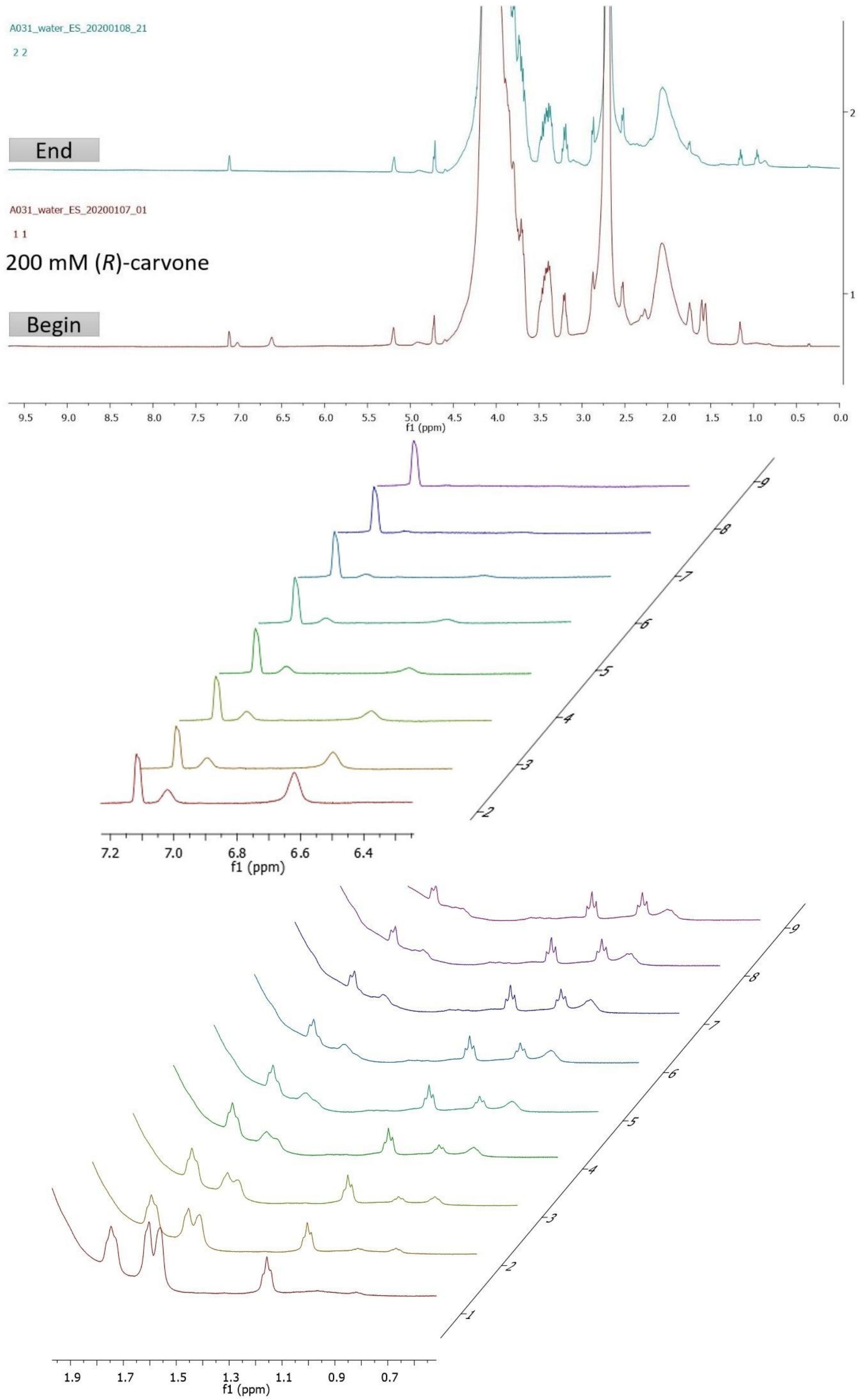

Figure S25. ^{1}H NMR spectrum of 200 mM (*R*)-carvone reduction, full spectra; 7.2 to 6.3 ppm; 1.8 to 0.5 ppm.

To address the problem of diffusion, another experiment was done, this time with spinning. The substrate concentration was reduced to 50 mM, to shorten reaction duration. A reduced volume (300 µL) with 16 scans and a 20 Hz spinning, to promote mixing of the sample. Comparison between the deuterated benzene peaks between the two experiments showed a large variation of peak areas with spinning (**Figure S26**), such that the spectra data with the 20 Hz spinning was unreliable.

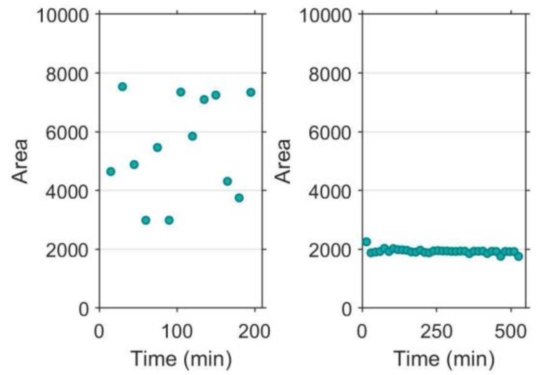

Figure S26. The effect of spinning on ^{1}H NMR integrals. **Left:** integral area of standard C_6D_6 peaks with 20 Hz spinning on a ^{1}H NMR with reaction conditions: 20 Hz spinning 0.5 mL total volume, 50 mM (*R*)-carvone, 4% v/v DMSO, 200 mM glucose, 3 U/mL *Bs*GDH, 0.1 mM NADP^{+}, 200 mM KPi pH 7.0. **Right:** integral area of standard C_6D_6 peaks without spin on a ^{1}H NMR. Reaction conditions: 200 mM (*R*)-carvone; 1 mL, 17% v/v DMSO, 210 mM glucose, 3 U/mL *Bs*GDH, 0.1 NADP^{+}, 2 µM *Ts*OYE, 200 mM KPi pH 7.0. The same closed capillary with C_6D_6 for locking was used for both reactions, with the water_ES pulse program, 64 scans at 30 °C with 15 min interval between measurements.

Another experiment was run, this time with a different NMR program. The first two experiments used NMR program Water_ES, which scouted for water peaks and suppressed them for each run, doubling the measuring time. In this experiment we used the presat purge program, which enabled us to select the water peak (range) that was to be suppressed. The 50 mM of substrate concentration was kept, also the 16 scans. The resulting integral areas from multiple chemical shift intervals that had shifted were calculated and plotted against time (**Figure S27**). Each graph is numbered and represents a chemical shift range. Some ranges are shown as a control peak, unchanged during the reaction (internal standard, and buffer peak). Other peaks that were declining in slope (substrate being converted) and some were increasing (product being formed). After 30 min most of the integral areas had stabilized.

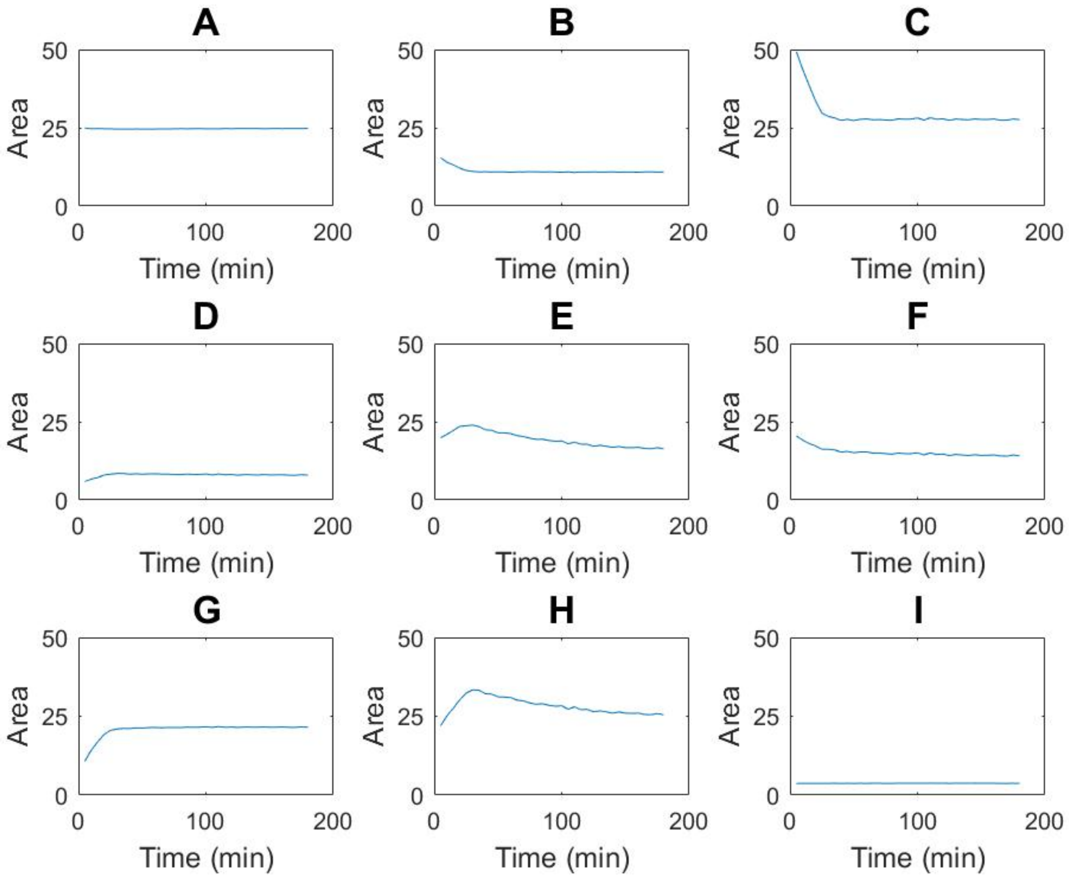

Figure S27. ^{1}**H NMR spectra peak integral areas over time. A)** 7.12 to 7.07 ppm representing the deuterated benzene standard in closed capillary. **B)** 6.98 to 6.92 ppm. **C)** 1.71 to 1.65 ppm. **D)** 1.62 to 1.59 ppm. **E)** 1.60 to 1.54 ppm. **F)** 1.55 to 1.48 ppm. **G)** 0.93 to 0.88 ppm. **H)** 0.86 to 0.75 ppm. **I)** 0.34 to 0.33 ppm. The shift in curves (B-H) occurred at the ~30 min mark where initial slopes from minute 0 to 30 are: A is -0.01, B is -0.17, C is -0.83, D is 0.10, E is 0.16, F is -0.17, G is 0.41, H is 0.46 and I is 0.00.

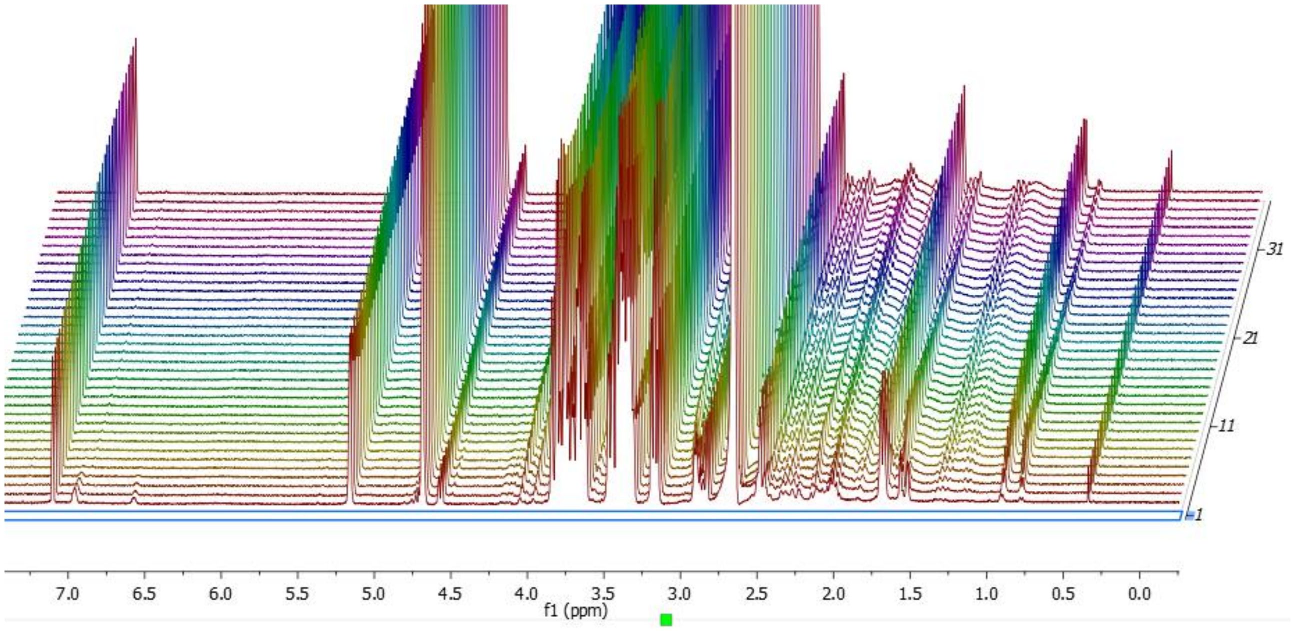

Figure S28. Full ^{1}H NMR spectrum of the 50 mM (*R*)-carvone reduction experiment, peak at 7.16 ppm is benzene-d_6 as reference.

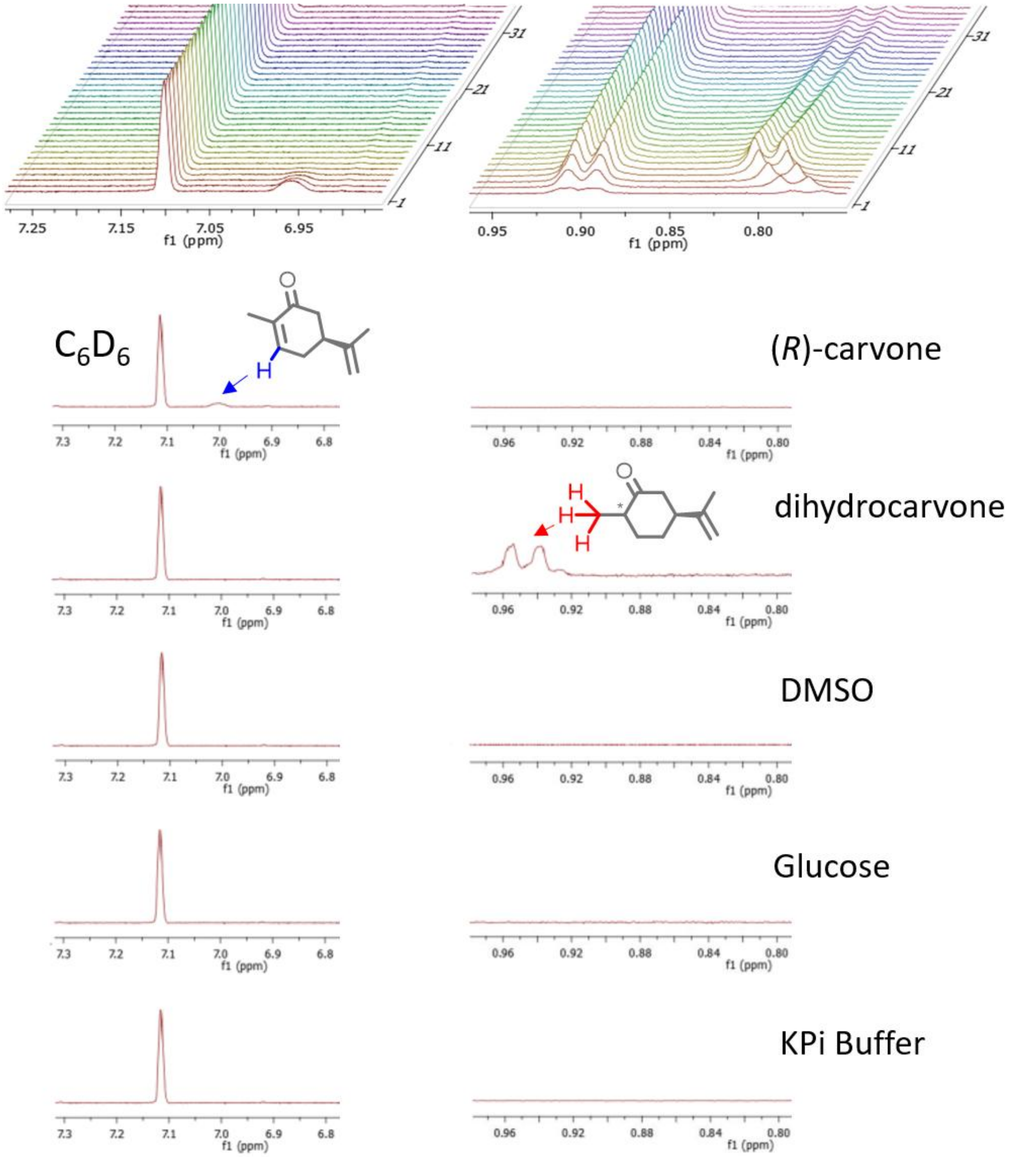

Figure S29. **^{1}H NMR monitoring of *Ts*OYE-catalyzed (*R*)-carvone reduction.** Two peaks were monitored: substrate (*R*)-carvone singlet peak (~6.95 ppm) and (2*R*,5*R*)-dihydrocarvone doublet peak (~0.9 ppm). The standard deuterated benzene peak is shown with chemical shift 7.16 ppm. **TOP**: array ^{1}H NMR spectra of 200 mM KPi-HCl buffer pH 7.0, 3 U/mL *Bs*GDH, 55 mM glucose, 0.1 mM $NADP^+$, 50 mM (*R*)-carvone, 4% v/v DMSO, 2 µM *Ts*OYE, 300 µL volume. NMR program was presat purge, 16 scans. Measurement intervals every 5 min. **BOTTOM**: ^{1}H NMR reference spectra of major reaction components, with standard using water_ES program, 8 scans, at 25 °C.

Figure S29 gives an overview of the two main peaks that were followed, at 6.98 and 0.92 ppm, as well as the standard deuterated benzene at 7.16 ppm. The singlet peak at 6.98 ppm represents the proton from the β-carbon of the double bond on the substrate (*R*)-carvone, while the doublet peak at 0.92 ppm represents the protons from the methyl group on the α-carbon of the product (2*R*,5*R*)-dihydrocarvone. The array spectra clearly show the declining peak of the substrate, the increasing peak of the product, as well as a product peak appearing at chemical shift 0.8. This peak may relate to the enolisation of the product.

NMR is a valuable technique to characterize products but also to monitor a reaction *in situ*, during which intermediates can become visible. For the ^{1}H NMR kinetic study of 50 mM (*R*)-carvone reduced to (2*R*,5*R*)-dihydrocarvone by *Ts*OYE, the doublet peak at 0.8 ppm belongs to a compound that is neither the substrate nor the product nor any component added to the solution (**Figure S29**). We hypothesize this doublet peak may be an enolate formed from the product where C5 protons are involved, or possibly a diastereomer. The (*R*)-carvone reduction monitoring revealed an unidentified species that need further investigation.

The ^{1}H NMR experiment with 200 mM (*R*)-carvone showed substrate peaks disappearing over time, however, the post reaction GC chromatogram showed 43% conversion. This observation is ascribed to the poor water solubility of carvone in water, *ca.* 9 mM at 25 °C,[13] and therefore the unreacted substrate in solution was indistinguishable on the NMR spectrum. The ^{1}H NMR calibration line, where 1 mM formed a visible peak, shows that the fast disappearing peaks may be due, in part, to diffusion. Previous studies showed that ^{1}H NMR measurements of water soluble substrates do produce a linear calibration line.[14] Therefore, careful analysis is needed when using poorly water soluble substrates.

Cyclohexenone reduction

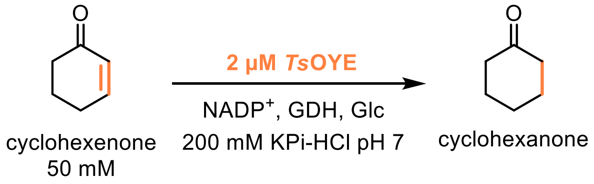

Reaction set-up

General stocks: 10 mM NADP+, 148 U/mL *Bs*GDH, 1 M glucose, 1 M cyclohexanone in DMSO, 60 µM *Ts*OYE. Reaction conditions: 200 mM KPi pH 7, 0.2 µM NADP+, 3 U/mL *Bs*GDH, 55 mM glucose, 50 mM cyclohexanone, 2 µM *Ts*OYE, 300 µL. All the reaction components were mixed in an Eppendorf tube except for *Ts*OYE, transferred to an NMR tube *Ts*OYE was added and the benzene-d_6 capillary. 16 scans were measured with the presat PURGE pulse sequence, no spin.

The array spectra show clear growth and shrinking of peaks (**Figure S30** and **Figure S31**). Compared with the experiment with 50 mM (*R*)-carvone, 50 mM cyclohexenone showed less signs of diffusion, and graphically shows full conversion with declining peaks have areas that move towards zero over time. The peak integration for the alkene α- and β-protons seems to indicate enolisation.

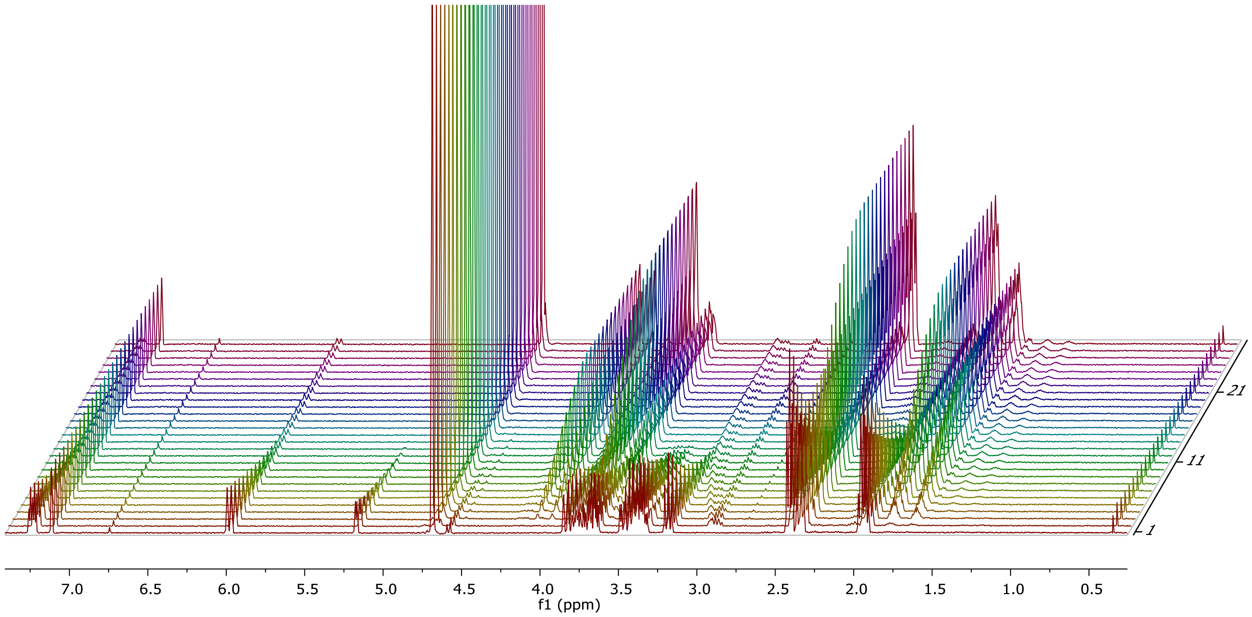

Figure S30. Full ^{1}H NMR spectrum of the cyclohexenone reduction experiment, peak at 7.16 ppm is benzene-d_6 as reference.

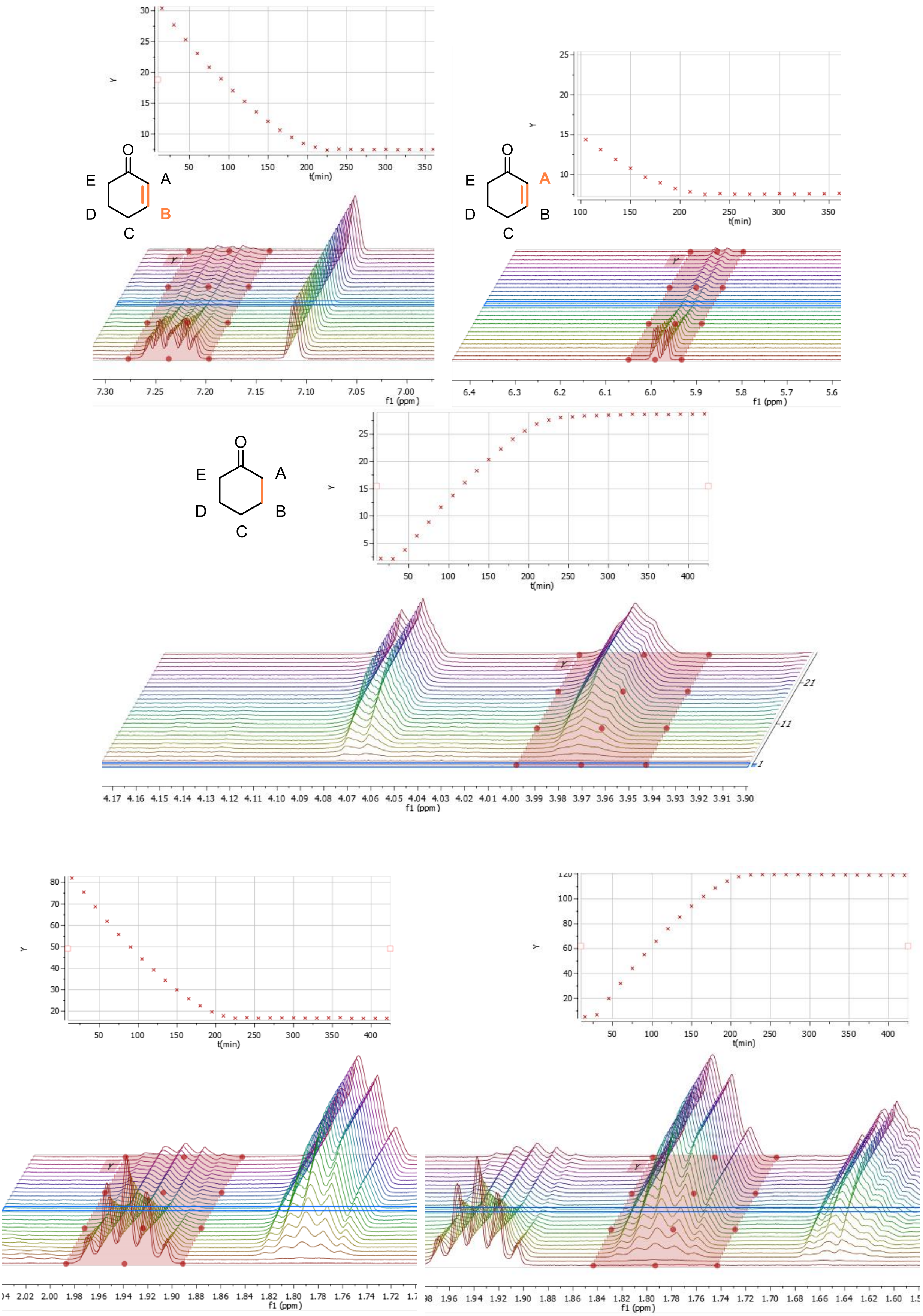

Figure S31. **^{1}H NMR monitoring of *Ts*OYE-catalyzed cyclohexenone reduction.** Growth and decline of peaks during the reaction. The integral areas plotted derived from array of ^{1}H NMR spectra. Conditions: 200 mM KPi-HCl buffer pH 7.0, 55 mM glucose, 0.2 mM NADP^{+}, 3 U/mL *Bs*GDH, 2 µM *Ts*OYE, 50 mM cyclohexenone, 300 µL volume. Measurement intervals every 5 min, with presat PURGE, 16 scans.

References

(1) Buque-Taboada, E. M.; Straathof, A. J. J.; Heijnen, J. J.; van der Wielen, L. A. M. *In situ* product removal using a crystallization loop in asymmetric reduction of 4-oxoisophorone by *Saccharomyces cerevisiae*. *Biotechnol. Bioeng.* **2004**, *86*, 795–800.

(2) Paul, C. E.; Gargiulo, S.; Opperman, D. J.; Lavandera, I.; Gotor-Fernández, V.; Gotor, V.; Taglieber, A.; Arends, I. W. C. E.; Hollmann, F. Mimicking nature: synthetic nicotinamide cofactors for C=C bioreduction using enoate reductases. *Org. Lett.* **2013**, *15*, 180–183.

(3) Knaus, T.; Paul, C. E.; Levy, C. W.; de Vries, S.; Mutti, F. G.; Hollmann, F.; Scrutton, N. S. Better than Nature: nicotinamide biomimetics that outperform natural coenzymes. *J. Am. Chem. Soc.* **2016**, *138*, 1033–1039.

(4) Guarneri, A.; Westphal, A. H.; Leertouwer, J.; Lunsonga, J.; Franssen, M. C. R.; Opperman, D. J.; Hollmann, F.; van Berkel, W. J. H.; Paul, C. E. Flavoenzyme-mediated regioselective aromatic hydroxylation with coenzyme biomimetics. *ChemCatChem* **2020**, *12*, 1368–1375.

(5) Wolder, A. E.; Heckmann, C. M.; Hagedoorn, P.-L.; Opperman, D. J.; Paul, C. E. Asymmetric monoreduction of α,β-dicarbonyls to α-hydroxy carbonyls by ene reductases. *ACS Catal.* **2024**, *14*, 15713–15720.

(6) Opperman, D. J.; Sewell, B. T.; Litthauer, D.; Isupov, M. N.; Littlechild, J. A.; van Heerden, E. Crystal structure of a thermostable Old Yellow Enzyme from *Thermus scotoductus* SA-01. *Biochem. Biophys. Res. Commun.* **2010**, *393*, 426–431.

(7) Macheroux, P. UV-visible spectroscopy as a tool to study flavoproteins. In *Flavoprotein Protocols*, Chapman, S. K., Reid, G. A. Eds.; Humana Press, 1999; pp 1–7.

(8) Sun, L.; Liu, Y.; Song, H.; Hao, J.; Lin, L. Engineering of an ene-reductase for producing the key intermediate of antiepileptic drug Brivaracetam. *Appl. Microbiol. Biotechnol.* **2023**, *107*, 1649–1661.

(9) González-Rodríguez, J.; González-Granda, S.; Kumar, H.; Alvizo, O.; Escot, L.; Hailes, H. C.; Gotor-Fernández, V.; Lavandera, I. BioLindlar catalyst: ene-reductase-promoted selective bioreduction of cyanoalkynes to give (*Z*)-cyanoalkenes. *Angew. Chem. Int. Ed.* **2024**, *63*, e202410283.

(10) Piotto, M.; Saudek, V.; Sklenář, V. Gradient-tailored excitation for single-quantum NMR spectroscopy of aqueous solutions. *J. Biomol. NMR* **1992**, *2*, 661–665.

(11) Hwang, T. L.; Shaka, A. J. Water suppression that works. excitation sculpting using arbitrary wave-forms and pulsed-field gradients. *J. Magn. Reson. Ser. A.* **1995**, *112*, 275–279.

(12) Simpson, A. J.; Brown, S. A. Purge NMR: Effective and easy solvent suppression. *J. Magn. Reson.* **2005**, *175*, 340–346.

(13) Fichan, I.; Larroche, C.; Gros, J. B. Water solubility, vapor pressure, and activity coefficients of terpenes and terpenoids. *J. Chem. Eng. Data* **1999**, *44*, 56–62.

(14) Marsden, S. R.; Gjonaj, L.; Eustace, S. J.; Hanefeld, U. Separating thermodynamics from kinetics-A new understanding of the transketolase reaction. *ChemCatChem* **2017**, *9*, 1808–1814.
